# Effect of gut hormones on bone metabolism and their possible mechanisms in the treatment of osteoporosis

**DOI:** 10.3389/fphar.2024.1372399

**Published:** 2024-04-25

**Authors:** Hongyu Liu, Huimin Xiao, Sufen Lin, Huan Zhou, Yizhao Cheng, Baocheng Xie, Daohua Xu

**Affiliations:** ^1^ Guangdong Key Laboratory for Research and Development of Natural Drugs, Dongguan Key Laboratory of Traditional Chinese Medicine and New Pharmaceutical Development, School of Pharmacy, Guangdong Medical University, Dongguan, China; ^2^ Institute of Traditional Chinese Medicine and New Pharmacy Development, Guangdong Medical University, Dongguan, China; ^3^ Department of Pharmacy, The 10th Affiliated Hospital of Southern Medical University (Dongguan People’s Hospital), Dongguan, China

**Keywords:** GLP-1, GLP-2, GIP, PYY, gut–bone axis, bone metabolism, gut microbiota

## Abstract

Bone is a highly dynamic organ that changes with the daily circadian rhythm. During the day, bone resorption is suppressed due to eating, while it increases at night. This circadian rhythm of the skeleton is regulated by gut hormones. Until now, gut hormones that have been found to affect skeletal homeostasis include glucagon-like peptide-1 (GLP-1), glucagon-like peptide-2 (GLP-2), glucose-dependent insulinotropic polypeptide (GIP), and peptide YY (PYY), which exerts its effects by binding to its cognate receptors (GLP-1R, GLP-2R, GIPR, and Y1R). Several studies have shown that GLP-1, GLP-2, and GIP all inhibit bone resorption, while GIP also promotes bone formation. Notably, PYY has a strong bone resorption-promoting effect. In addition, gut microbiota (GM) plays an important role in maintaining bone homeostasis. This review outlines the roles of GLP-1, GLP-2, GIP, and PYY in bone metabolism and discusses the roles of gut hormones and the GM in regulating bone homeostasis and their potential mechanisms.

## 1 Introduction

Research on incretin hormones has never stopped. The term “incretin” was originally coined by Creutzfeld (Creutzfeldt, 1979) in 1979 to represent the hormone secreted by the gut that stimulates the release of insulin in a glucose-dependent manner and is a critical regulator of energy metabolism. Although several insulin-stimulating hormones are secreted in the gut, there are only two physiological incretins so far, including glucagon-like peptide-1 (GLP-1) and glucose-dependent insulin-stimulating peptide (GIP). It was later discovered that these two hormones mediate the gut–bone axis and have a role in regulating bone metabolism. Glucagon-like peptide-2 (GLP-2), which is co-secreted with GLP-1, also regulates bone metabolism and is also part of the gut–bone axis. Several investigations, including human trials (Baggio and Drucker, 2007; Bergmann et al., 2019; Gabe et al., 2022; Gaudin-Audrain et al., 2013; Kreitman et al., 2021; Luo et al., 2016; Ma et al., 2013; Mabilleau et al., 2016; Mieczkowska et al., 2013; Nissen et al., 2019; Skov-Jeppesen et al., 2021; Westberg-Rasmussen et al., 2017), have elucidated that three hormones comprehensively suppress the process of bone resorption, with an added virtue of GIP fervently stimulating bone formation. Another incretin hormone, peptide YY (PYY), which is also secreted along with GLP-1 and GLP-2, has the most prominent role in appetite suppression, but recent studies have also shown that PYY has a strong inhibitory effect on bone formation (Jensen et al., 2021; Leitch et al., 2019; Russell et al., 2009; Scheid et al., 2011; Wong et al., 2012; Wortley et al., 2007). Notably, with increasing research on the gut microbiota (GM) in recent years, it has been found that the GM plays an important role in the regulation of bone metabolism, and the GM regulates bone homeostasis in a variety of ways, including influencing host metabolism, participating in immune regulation, and influencing endocrine bone signaling factors (including influencing the secretion of gut hormones). Herein, we review the relationship, potential mechanisms, clinical application prospects, and challenges of gut hormones, the GM and osteoporosis in the light of emerging literature and based on relevant studies in order to update the knowledge in this research area and to provide some references for future related studies.

### 1.1 Bone

Bone, as a highly dynamic organ, is extremely important for the human body. It provides rigidity and shape, supports the body structure, protects the vital organs, and aids locomotion. More importantly, as an endocrine organ, bone also participates in the metabolic processes of the human body. As the storehouse of calcium and phosphorus, bone tissue, along with the intestines and kidneys, is of great significance in maintaining the metabolic balance of phosphate and calcium ions in the body (Hill Gallant and Spiegel, 2017; Jouret et al., 2013).

Bone tissue consists of cellular components in an extracellular matrix. Bone-lining cells, osteoblasts, osteoclasts, and osteocytes are composed of cellular components. Their activity is regulated by mechanical forces, cytokines, hormones (e.g., parathyroid hormone (PTH)), bone cell turnover, and local factors. The differentiation of mesenchymal stem cells (MSCs) depends on the Wnt/β-catenin pathway. Under the regulation of transcription factors such as runt-related transcription factor 2 (RUNX2) and osterix (OSX), MSCs differentiate into osteoblast precursors and, eventually, osteoblasts. Sclerostin encoded by SOST (a soluble molecule that binds to low-density lipoprotein receptor-related protein-5/6 (LRP5/6)) can inhibit the Wnt signaling pathway by competitively inhibiting the coreceptor LRP5/6 in the signal pathway, thereby inhibiting osteoblast differentiation (Krause et al., 2010). Immature osteoblasts activate intracellular protein kinase A (PKA), protein kinase C (PKC), and calcium signal pathways under PTH signaling stimulation, induce osteoclast activation and differentiation, and then establish bone resorption (Rendina-Ruedy and Rossen, 2022). On the other hand, mature osteoblasts are produced and buried in matrix proteins (collagen) and are referred to as osteocytes. Osteocytes account for the majority of cells in mature mineralized bone; they have many linear pseudopods that communicate with osteoblasts, inactive bone-lining cells, and osteoclasts that are recruited on the bone surface to form a network of interconnections. Osteocytes secrete sclerostin in response to mechanical stress, which acts on the Wnt/β-catenin pathway in osteoblasts to inhibit cell proliferation, impair mineralization, enhance apoptosis, and promote osteoclast formation and activity in a RANKL (receptor activator of nuclear factor κB (NF-κB) ligand)-dependent manner (Horwood, 2016). Therefore, sclerostin produced by osteocytes regulates both osteoclasts and osteoblasts. Osteoclasts, the sole bone-resorbing cells, are multinucleated cells tightly attached to the surface of bone, are derived from the macrophage/monocyte lineage, and attach to the bone through a sealing zone where they secrete acids and proteases that break down the mineral and organic phases of bone in a spatially controlled manner. Macrophage colony-stimulating factor (M-CSF) and RANKL are key cytokines required for the survival, expansion, and differentiation of osteoclast precursor cells *in vitro* (Raggatt and Partridge, 2010). When RANK on the surface of osteoclast precursor cells binds with RANKL released by osteoblasts, the expression of osteoclast-related genes in the nucleus increases through the NF-κB and calmodulin/nuclear factor of activated T-cell (CN/NFATc1) pathways, which ultimately results in the activation of osteoclast-related genes in the osteoclast precursor cells and thus promotes osteoclastic differentiation. Mice lacking functional M-CSF or RANKL genes cannot absorb bone, resulting in ossification (Kim et al., 2020; Salhotra et al., 2020). Osteoprotegerin (OPG) is both a soluble bait receptor of RANKL and a negative physiological regulator of osteoclast formation that can effectively block the binding of RANKL to RANK on osteoclast precursors, delay the activation of osteoclasts, and inhibit bone resorption. Current studies have shown that the proportion of OPG/RANKL expression determines the differentiation and function of osteoclasts (Yang et al., 2020). Downregulation of the OPG/RANKL ratio causes animals to develop osteoporosis due to excessive osteoclastogenesis.

### 1.2 Bone remodeling

**FIGURE 1:**
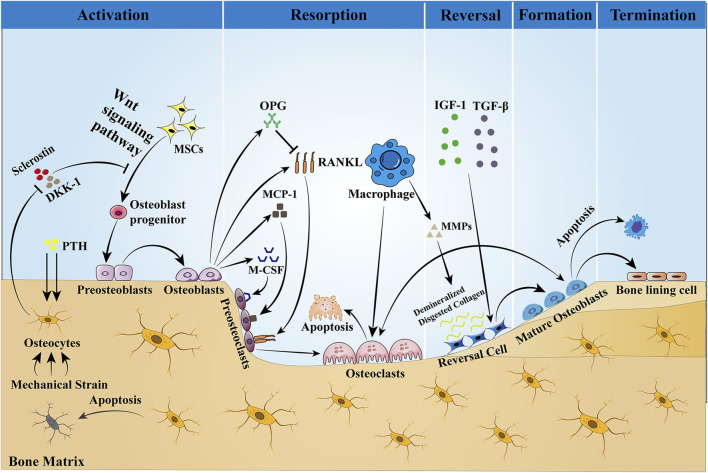
Bone remodeling is a dynamic process that is completed with the participation of BMU. This process is regulated by local intercellular signals and external signaling factors. The cellular components of BMU include osteoblasts, reversal cells, and bone-lining cells. Osteoblasts are derived from MSCs, a process that is dependent on the Wnt pathway and regulated by the transcription factors Runx2 and OSX. Sclerostin is a negative regulator of Wnt, which prevents mesenchymal stem cells from differentiating into osteoblasts. When the bone is stimulated by mechanical traction or PTH, the intracellular PKA and PKC pathways are activated, which initiates bone remodeling by inhibiting sclerostin expression and activating the Wnt signaling pathway. Osteoblasts stimulate osteoclast precursors to differentiate into mature osteoclasts by secreting M-CSF, MCP-1, and RANKL, but they may also inhibit the same cells by secreting OPG that scavenges RANKL, preventing it from binding to the RANKL receptors on the osteoclast precursors. Then, bone resorption begins, producing Howship’s resorption lacunae. After bone matrix resorption, macrophages produce MMPs to degrade and remove the bone matrix, and the release of active TGF-β and IGF-1 triggers osteoblasts to form a new collagen matrix in Howship’s resorption lacunae and fill the absorption space. Finally, the remodeling cycle ends when an equal amount of resorbed bone is replaced. In the bone reconstruction of healthy people, there was no change in bone mass and strength at the end of each reconstruction cycle. However, under some pathological conditions, such as osteoporosis, both bone mass and strength are decreased.

Bone, a highly dynamic organ, continues to remodel throughout life to help maintain bone strength and function, adapt to the body’s changing mechanical needs, and maintain calcium–phosphorus balance within the body. Healthy bone must be maintained through ongoing skeletal remodeling in order to perform these critical functions throughout life. Bone remodeling is a physiological process that involves the coordinated activity of a group of cells known as the basic multicellular unit (BMU) (Buenzli et al., 2014). The intrinsic communication within the BMU is frequently depicted as an intricate network of regulation among distinct cellular constituents, whereby the osteocyte population regulates the activity of the osteoblast population, and the osteoblast population, in turn, regulates the activity of the osteoclast population. A complete BMU consists of bone-lining cells that regulate the activity of osteoclasts, osteoblasts that are responsible for bone resorption, reversal cells that are responsible for transition, and mature osteoblasts that are responsible for mineralization, which coordinate their activities with each other in time and space to ensure the orderly progression of the entire process of bone reconstruction: activation, resorption, reversal, formation, and termination, a process that will be discussed below and illustrated schematically in Figure 1.

Activation Phase: In the resting state, osteocytes secrete TGF-β, which inhibits osteoclast formation. When osteoblasts are subjected to mechanical loading or when microdamage is detected in old bone (Bonewald, 2007), the signals that inhibit osteoclastogenesis are eliminated and osteoblasts are allowed to be generated (Jann et al., 2020). In addition, PTH signaling activates bone remodeling by inducing osteoblasts to secrete regulatory molecules that recruit osteoclast precursors to specific resorption sites.

Resorption phase: Osteoblasts play a crucial role in establishing bone resorption. As a response to PTH-induced bone remodeling, osteoblasts produce the chemokine monocyte chemotactic protein-1 (MCP-1) *in vivo*, which is a chemokine for osteoclast precursors and promotes RANKL-induced osteoclast formation *in vitro* (Li et al., 2007). In addition to the re-recruitment of osteoclast precursors, the major osteoclast factors expressed by osteoblasts, M-CSF, and RANKL are involved in the regulation of bone resorption. M-CSF and RANKL promote the proliferation and survival of osteoclast precursor cells and additionally coordinate the differentiation of osteoclast precursors into multinucleated osteoclasts to promote resorptive activity (Boyce, 2013; Burgess et al., 1999). Matrix metalloproteinases (MMPs) secreted by osteoblasts degrade unmineralized bone-like material arranged on the bone surface, which in turn promotes osteoclast attachment to the RGD-binding site, where the mineralized matrix is dissolved to form the Howship lacuna (Feng and McDonald, 2011). Some of the remaining undigested organic bone matrix is subsequently degraded by collagenases (especially histone K) (Lu et al., 2018).

Reversal phase: Following osteoclast-mediated resorption, the Howship lacunae are covered with a partially digested demineralized collagen matrix (Saftig et al., 1998). To eliminate these collagen remnants and prepare the bone surface for subsequent bone formation orchestrated by osteoblasts, early reversal cells exhibit a pro-resorptive phenotype and secrete matrix metalloproteinases that help osteoclasts to open channels for mineralized bone matrix and degrade the upper layer of non-mineralized collagen. In addition, reversal cells produce RANKL to aid in osteoclast resorption. In addition to reversing cells, macrophages can also produce MMPs, the enzymes required for matrix degradation, and are professional phagocytic cells. Late-differentiated reversed cells with fewer vesicles become mature osteoblasts with a pro-synthesizing phenotype and produce various cytokines such as OPG, which inhibit the proliferation and differentiation of osteoclasts and promote their apoptosis, thus playing an important role in adult bone reconstruction (Abdelgawad et al., 2016). Thus, the ultimate function of reversal cells may lie in their ability to receive or generate coupled signals that allow the transition from bone resorption to bone formation within the BMU.

Formation phase: In the stage of bone formation, many reversal cells close to osteoid proliferate and differentiate into mature osteoblasts, which produce osteoid and are responsible for mineral deposition. Meanwhile, both the insulin-like growth factor-1 (IGF-1) released during bone resorption and TGF-β are key signals for MSC recruitment to the site of bone resorption, and their expression promotes the return of MSCs or early osteoblast precursor cells to the resorption traps, ultimately allowing them to differentiate and secrete the molecules that form the replacement bone (Tang et al., 2009). Finally, to give the bone its full shape, hydroxyapatite is incorporated into this newly deposited bone-like material, ultimately forming high-quality mineralized bone and transitioning bone reconstruction to the termination stage.

Termination phase: The remodeling process concludes once an equal quantity of resorbed bone has been replaced. Sclerostin that inhibits bone formation is re-expressed at the end of remodeling, allowing bone mass to remain stable. Upon completion of mineralization, mature osteoblasts are transformed into bone-lining cells covering the bone surface or embedded in the mineralized matrix and are designated as bone cells. The quiescent bone surface environment is rebuilt and maintained until the next wave of remodeling begins.

Such a dynamic remodeling process is essential for bone self-homeostasis and for maintaining skeletal strength and calcium–phosphorus homeostasis (Siddiqui and Partridge, 2016). In the process of bone reconstruction in healthy people, there was no net change in bone mass and strength after each reconstruction cycle. However, bone mass and strength are reduced under pathological conditions, such as bone remodeling disorders in patients with osteoporosis.

### 1.3 Bone turnover markers

The International Osteoporosis Foundation and the International Federation of Clinical Chemistry recommend serum type I collagen C-terminal peptide (CTX-I) and type I procollagen N-terminal propeptide (P1NP) as two reference markers (Vasikaran et al., 2011). All research on bone metabolism is supposed to include at least these two markers. CTX-I is a part of the cross-linker decomposed by type I collagen, which is derived only from mature type I collagen, is not degraded or reutilized *in vivo*, and enters the blood at the time of bone resorption, which directly reflects the degradation of collagen fibers of the bone and decreases during antiresorptive therapy. Therefore, circulating CTX-I levels are used as biomarkers of bone resorption. P1NP originates from type I pre-collagen and is cleaved by protease after translation. Except for a small amount of P1NP deposited in the bone matrix, a large amount of it enters the blood circulation; thus, serum pre-collagen is mainly derived from bone and serves as a marker of bone formation (Schiellerup et al., 2019). Because multiple factors influence the levels of bone formation markers in the body, including hormone levels and body weight (Eastell et al., 2012; Evans et al., 2015), the introduction of a monitoring program is of great importance in determining the validity of bone conversion markers and also contributes to the reduction of fracture risk. However, conducting an extensive and comprehensive long-term study is imperative in order to obtain reliable results. Therefore, researchers may need to accept a degree of uncertainty.

## 2 Gut–bone axis

The gut and bone are interconnected to form a gut–bone axis, and this interaction is regulated by hormones secreted from the gut. These hormones, which are secreted in response to nutritional intake, lead to decreasing bone resorption (Clowes et al., 2002; Henriksen et al., 2003; Westberg-Rasmussen et al., 2017). Throughout the day, bone is remodeled, absorbed and formed in a coupled process to maintain dynamic balance (Eastell and Szulc, 2017; Zaidi et al., 2018). Eating during the day inhibits bone resorption but increases bone resorption at night, and this day–time suppression is eliminated by fasting, further affirming the role of the gut hormones in the control of bone homeostasis. The gut is one of the most important endocrine organs in the human body, from which many hormones are secreted. Among many hormones, a class of peptides known as incretin hormones has become an important regulator of energy metabolism. “Incretin hormones” are an endocrine signal secreted by the gut after eating that stimulates insulin release in a glucose-dependent manner (Creutzfeldt, 1979). Although the gut secretes a variety of insulin-stimulating hormones, it is currently believed that only GLP-1 and GIP are glucose-dependent insulin secretion stimulators, while other gut hormones, such as GLP-2 and PYY, may indirectly stimulate insulin secretion by increasing blood glucose.

The effects of GIP (secreted by enteroendocrine K-cells) and GLP-1 (secreted by L-cells) on glucose metabolism as mediators of the insulin effect have been extensively studied: enhanced insulin secretion occurs when glucose is ingested orally (Drucker, 2018). As a result, there has been a strong interest in their use in the treatment of type 2 diabetes mellitus (T2DM) and obesity. Many of the drugs widely used in the treatment of T2DM, such as liraglutide and exenatide, are GLP-1 receptor agonists (GLP-1RAs). By promoting postprandial insulin secretion, these hormones have the potential to elicit insulin-mediated enhancements in bone formation. Although short-term insulin injections during normoglycemia have essentially no effect on circulating levels of bone turnover markers (Frost et al., 2018), it is unclear whether long-term increases in insulin levels modulate insulin-mediated increases in bone formation. GLP-2, along with GLP-1, is released from the L-cells of the small intestine, and unlike the hypoglycemic effects of GLP-1 and GIP, GLP-2 is considered more of a pro-intestinal factor, which has led to the development of GLP-2 receptor agonists (GLP-2RAs) as therapeutic agents for short bowel disease (Drucker et al., 1996; Tsai et al., 1997). Unlike GLP-1, the role of GIP in bones is insulin-independent (Christensen et al., 2018). Another hormone, PYY, is similarly secreted along with GLP-1 and GLP-2 in PP cells and may affect bone metabolism by inhibiting bone formation. GLP-1, GLP-2, and GIP are considered to act directly or indirectly on bone cells to prevent bone resorption, while PYY inhibits bone formation. All four incretin hormones are associated with the gut–bone axis and will be summarized in Table 1.

**TABLE 1:** Summary of the known effects of gut hormones on bone homeostasis.

Hormones	Subjects and design	Species	Main results	References
GLP-1	*In vitro*	Human	The presence of GLP-1R has been detected in certain osteoblastic cell lines. GLP-1 was found to promote the osteogenic differentiation process of human BMSCs and inhibit their tendency toward adipogenic differentiation. It reduces cell death, protects bone loss and fracture, and promotes bone formation.	Lu et al. (2015), Meng et al. (2016), Pacheco-Pantoja et al. (2016), Pacheco-Pantoja et al. (2011)
		Mouse	The presence of GLP-1R has been detected in mouse osteoblast-like cells. In numerous studies, GLP-1 has been found to play a role in promoting osteoblast differentiation and proliferation through the classical cAMP/PKA/β-CAT-Ser675, PI3K, and MAPK pathways, as well as exerting some effects on osteoclasts.	Aoyama et al. (2014), Feng et al. (2016), Hu et al. (2016), Pereira et al. (2015), Wu et al. (2017)
		Rat	The presence of GLP-1R has been detected in rat osteoblasts and osteocytes. GLP-1 exerts a regulatory effect on osteoblast differentiation and regulates osteoclast protein synthesis.	Kim et al. (2013), Lu et al. (2015)
	*In vivo*	Human	GLP-1 has a beneficial effect on bone metabolism, possibly through the promotion of bone formulation. It has no effect on bone turnover markers or BMD. Meta-analysis shows that GLP-1RAs (liraglutide and exendin-4) have an effect on the risk of fracture. Liraglutide can significantly reduce the risk of fracture, while exendin-4 can increase the risk of fracture, but there was no significant effect in patients with T2DM.	Bunck et al. (2011), Christensen et al. (2018), Driessen et al. (2015), Driessen et al. (2015), Gilbert et al. (2016), Henriksen et al. (2003), Iepsen et al. (2015), Li et al. (2015), Mabilleau et al. (2014), Mabilleau et al. (2016), Su et al. (2015), Sun et al. (2015)
		Mouse	GLP-1R knockout mice have reduced tibial and vertebral cortical bone volume and strength and a significantly immature collagen matrix. Treatment with GLP-1RAs has been shown to confer protective effects against bone loss induced by ovariectomy (OVX) or diabetes.	Yamada et al. (2008), Mabilleau et al. (2013), Mansur et al. (2015), Mieczkowska et al. (2015), Pereira et al. (2015)
		Rat	GLP-1 treatment has a positive effect on promoting bone strength and enhancing bone quality and is effective in preventing bone loss. GLP-1RA dose-dependently increases femoral and lumbar BMD, improves trabecular structure, and decreases trabecular spacing in the femur and lumbar spine in OVX rats.	Kim et al. (2013), Lu et al. (2015), Ma et al. (2013), Meng et al. (2016), Nuche-Berenguer et al. (2011), Nuche-Berenguer et al. (2009), Nuche-Berenguer et al. (2010), Sun et al. (2016), Sun et al. (2015)
GLP-2	*In vitro*	Human	The presence of GLP-2R has been detected in MG-63 and TE-85 cell lines. GLP-2 promotes cell viability but does not affect ALP secretion in MG-63 and TE-85 cell lines.	Pacheco-Pantoja et al. (2011)
		Mouse	No research yet.	
		Rat	No research yet.	
	*In vivo*	Human	GLP-2 dose-dependently inhibits bone resorption (measured as CTX-Ⅰ) with only minimal effects on bone formation (measured as OCN or P1NP) in healthy postmenopausal women. Four months of GLP-2 treatment increases hip BMD in postmenopausal women with bone loss. GLP-2RA (teduglutide) increased total body BMC but not lumbar spine or hip BMD in SBS patients.	Henriksen et al. (2003), Henriksen et al. (2004), Henriksen et al. (2009), Jeppesen et al. (2011), Askov-Hansen et al. (2013), Skov-Jeppesen et al. (2019)
		Mouse	GLP-2 increased the bone mineral density, improved the microstructure of the femur, enhanced the osteogenic activity, and reduced the bone loss in SAMP6 mice but had no significant inhibitory effect on the activity of osteoclasts.	Huang et al. (2023)
		Rat	GLP-2 significantly increases BMD in the spinal region of SBS rats.	Scott et al. (1998)
GIP	*In vitro*	Human	The presence of GIPR has been detected in human bone marrow mesenchymal stem cells (hBMSCs) as well as human osteosarcoma cell lines (Saos-2, TE-85, and MG-63). GIP reduces osteoclast formation and resorption. In osteoblastic cell lines, GIP increase stimulates P1NP and ALP and promotes osteoblast proliferation and differentiation but diminishes cell death. GIP analogs reduce the differentiation and bone resorption activity of human osteoclasts.	Bollag et al. (2000), Pacheco-Pantoja et al. (2011), Berlier et al. (2015), Mabilleau et al. (2016)
		Mouse	GIP plays a role in inhibiting PTH-induced osteoclast resorption and also promotes ALP production and mineralization processes.	Ding et al. (2008), Zhong et al. (2006)
		Rat	No research yet.	
	*In vivo*	Human	GIP has an inhibitory effect on bone resorption and possibly promote osteosynthesis. GIP reduces CTX-Ⅰ independently of insulin. Loss-of function GIPR gene polymorphism has been found to be inversely associated with BMD and positively associated with an elevated risk of fractures. The antiresorptive effects of GIP are preserved in hypoparathyroid patients, supporting that GIP has PTH-independent effects on bone.	Bergmann et al. (2019), Christensen et al. (2018), Gasbjerg et al. (2020), Helsted et al. (2020), Nissen et al. (2014), Polymeris et al. (2011), Skov-Jeppesen et al. (2021), Torekov et al. (2014), Westberg-Rasmussen et al. (2017)
		Mouse	Bone formation parameters (e.g., BMD, bone trabecular volume, ALP, and OCN) showed a decreasing trend in GIPR knockout mice, whereas bone resorption parameters (e.g., CTX-Ⅰ and urinary deoxypyridine) showed an increasing trend. However, the results of different studies are not always in complete agreement.	Xie et al. (2005), Tsukiyama et al. (2006), Gaudin-Audrain et al. (2013), Mieczkowska et al. (2013)
		Rat	GIP improves spinal bone density and cortical bone properties in OVX rats.	Bollag et al. (2001), Baldassano et al. (2019)
PYY	*In vitro*	Human	No research yet.	
		Mouse	Y1R is expressed in mouse BMSCs and osteoblasts. PYY signaling in osteoblasts functions via the Y1 receptor. PYY has an antagonistic effect on osteoblast activity. Overexpression of PYY decreased osteoblasts and increased osteoclast activity.	Wong et al. (2012)
		Rat	Y1R antagonist improves bone microarchitecture and reduces bone microdamage in OVX rats.	Xie et al. (2020)
	*In vivo*	Human	PYY has a strong inhibitory effect on bone formation. Decreased PYY secretion in obese patients leads to increased BMD; increased PYY secretion in anorexia nervosa patients and after gastric bypass surgery leads to decreased BMD. Plasma PYY was negatively correlated with BMD (in anorexia nervosa patients and premenopausal athletic women) and with P1NP (in amenorrheic young female athletes).	Remmel et al. (2015), Russell et al. (2009), Scheid et al. (2011), Utz et al. (2008), Yu et al. (2016)
		Mouse	In transgenic mice, overexpression of PYY reduced bone mass, whereas YR1 deficiency increased osteoblast activity. Despite the controversy, PYY KO mice show increased bone mass and bone strength. The modulation of bone formation and resorption seems to be achieved via Y1, Y2, and Y6 receptors.	Khor et al. (2016), Lee et al. (2010), Lee et al. (2011), Wong et al. (2012), Wortley et al. (2007)
		Rat	Inhibition of Y1R signaling improves bone microarchitecture in OVX rats by increasing bone formation and decreasing bone resorption.	Wong et al. (2012), Xie et al. (2020)

**Summary:** GLP-1 acts directly on osteoblasts to increase formation and decrease resorption to affect bone mass and quality. GLP-2 has little effect on bone formation; as a result, it increases bone density primarily by inhibiting bone resorption. GIP directly regulates bone metabolism with anabolic effects on osteoblasts and antiresorptive effects on osteoclasts. PYY negatively regulates bone strength and bone mass in adults and has long-term harmful side effects on bone, including an increased risk of fractures.

## 3 Gut microbiota (GM)

A huge number of microorganisms are attached to the surface and body of the human and play a vital role in the physiological activities and pathological coordination of the body (Cryan et al., 2019). Of the trillions of microorganisms in the human body, the vast majority comprise the more than 1,000 species of GMs found in the gut (Rizzoli, 2019). The gut micro-ecosystem is the largest micro-ecosystem in the human body, with 1,014 micro-organisms known to date and a total number of genes approximately 150 times the number of genes in the human genome (Qin et al., 2010; Zhang et al., 2022). Under normal circumstances, the GM can establish a dynamic ecological balance with the host and the external environment that is conducive to the maintenance of human health (Pröbstel et al., 2020). However, the negative impacts on the GM caused by poor dietary habits, radiation therapy, the misuse of antibiotics in medical treatment, and changes in the living environment may lead to microbial imbalance and metabolic imbalance, which may induce a series of diseases, including various forms of enteritis, diabetes, obesity, rheumatoid arthritis, osteoporosis, and depression (Fava et al., 2019; Jian et al., 2021; Tap et al., 2021; Wang et al., 2021; Zhao et al., 2021). Related studies have shown that regulating the biological abundance of the GM can modulate the onset and progression of diseases, and thus the GM is a potential new target that may offer new therapeutic options for the treatment of metabolic diseases (Tanabe et al., 2019).

**FIGURE 2:**
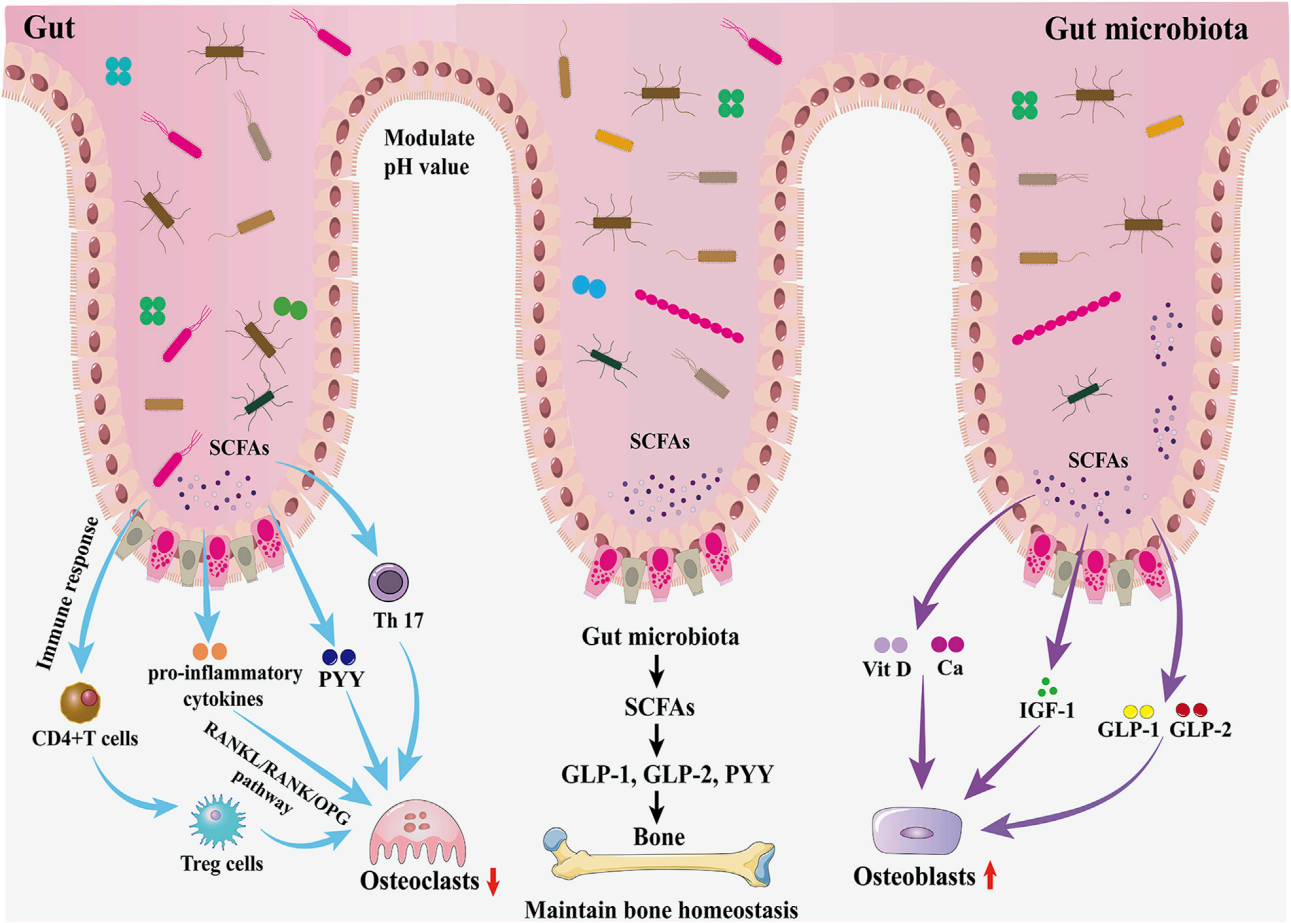
Effects of the GM and gut hormones on bone metabolism. The GM regulates bone homeostasis through multiple pathways, including host metabolism, immunomodulation, and hormonal regulation. On the one hand, the GM promotes the production of GLP-1 and GLP-2 by intestinal cells through SCFAs, thereby promoting bone formation; in addition, the increased secretion of IGF-1 is also favorable to bone formation. On the other hand, the GM inhibits osteoclast activity by inhibiting Th17 cell differentiation and thus inhibiting the release of RANKL.

Successive studies in recent years have shown that alterations in the GM are strongly associated with the onset and progression of osteoporosis (Chen et al., 2022; Guss et al., 2019; Nilsson et al., 2018). Due to the complexity of the mechanisms involved in the involvement of the GM in osteoporosis, some studies have been conducted in the context of host metabolism, immunity, and the endocrine environment (Behera et al., 2020; Iqbal et al., 2020) (Figure 2).

### 3.1 GM affects bone homeostasis through host metabolism

The GM may affect bone homeostasis through the metabolism of short-chain fatty acids (SCFAs). In the gut, lactose-based prebiotics are converted by probiotics into SCFAs (butyric, acetic, and propionic acids) and lactic acid (Borka Balas et al., 2023). They promote the synthesis of the gut hormones plasma GLP-1, GLP-2, and PYY by entero-endocrine L cells through the activation of GPCRs (Kasubuchi et al., 2015; Kellow et al., 2014). As described earlier, GLP-1 and GLP-2 play an important role in increasing the activity of osteoblasts and promoting bone formation. Therefore, the GM promotes bone formation through the increase of GLP-1 and GLP-2 secretion mediated by SCFAs. However, SCFA-induced increases in PYY secretion should not be overlooked because PYY has a strong bone resorption-promoting effect. IGF-1 is a hormone that can affect bone growth. It is one of the most abundant growth factors in bone, with receptors in both osteoblasts and osteoclasts, and can regulate the activity of osteoblasts, promote the formation of collagen, inhibit the degradation of collagen, and play a role in the maintenance of bone mass and bone density. The GM may maintain bone mass by increasing serum IGF-1 levels through the production of SCFAs. When SCFAs, a metabolite of GM, were supplemented to antibiotic-treated mice, IGF-1 and bone mass were restored to the levels of normal mice, suggesting that the restoration of the GM in germ-free mice induces circulating levels of IGF-1 through the production of SCFAs, which in turn affects bone metabolism (Yan et al., 2016). In addition, SCFAs can also lower the pH of the gut environment, prevent calcium ions from compounding with phosphorus to increase calcium absorption, and inhibit RANKL signaling pathway-induced osteoclastogenesis by suppressing osteoclast gene expression (Chen et al., 2020). Besides, the butyrate in SCFAs provides energy to the gut, repairs the structure of gut mucosa and villi, increases the absorption area of the gut, and increases the absorption and utilization of calcium in the gut.

### 3.2 GM affects bone homeostasis through the immune system

The GM is essential for the function and maturation of the immune system, which stimulates the immune system at the gut mucosal barrier through the release of metabolites and immune cells (including T cells and B cells), the release of pro- or anti-inflammatory mediators as well as cytokines, and the regulation of systemic bone metabolism via the blood circulation (Schluter et al., 2020). Activated immune cells can migrate to skeletal tissue and directly regulate bone metabolism by releasing products that include osteoclast-inducing factors such as RANKL, TNF-α, and interleukin (IL) (Pacifici, 2016). A study of the gut thick-walled *bacillus* phylum in mice showed that *Clostridium difficile* promotes the expression of helper T cells (Th cells) in the lamina propria of the colon. Th cells inhibit osteoclast differentiation and impede osteoblast formation, and a reduction in the number of *C. difficile* strains leads to lower levels of Th cells and an increase in bone turnover (Luo et al., 2011). The GM can also regulate the balance between intestinal Tregs and Th cells by influencing the development of Th cells and the differentiation of Tregs, thereby altering the internal environment of the gut and the body as a whole, regulating the function of osteoclasts and osteoblasts, and influencing the dynamic balance between bone resorption and bone formation (Sun et al., 2020). A GM imbalance may inhibit the differentiation of the Tregs subpopulation, leading to the predominant differentiation of Th17 cells, which belong to the CD4^+^T-cell osteoblastic cell group and can secrete IL-17a, IL-1, IL-6, tumor necrosis factor, and low levels of interferon-γ. These cytokines induce monocytes and macrophages to form osteoclasts and exacerbate bone loss by promoting the release of RANKL. Kim et al. (2017) transplanted a variety of commensal bacteria isolated from human feces into mice and found that the mouse gut segmented filamentous bacteria and human commensal bacteria promoted the differentiation of Th17 cells in the gastrointestinal tract of mice, which induced pro-inflammatory cytokines (including IL-17, TNF-α, and IL-1β), as well as the production of osteoclasts, and enhanced bone resorption.

### 3.3 GM affects bone homeostasis through hormones

Recent studies suggest that the GM may be involved in endocrine regulation of the body to alter the relative activity of osteoclasts and osteoblasts, thereby affecting bone metabolism and participating in the regulation of bone mass (Iqbal et al., 2020). Until now, the GM has been found to be strongly associated with the levels of several hormones, including gut hormones, IGF-1, PTH, and estrogen (Behera et al., 2020).

#### 3.3.1 GM affects bone homeostasis through IGF-1

The GM may regulate osteogenesis by affecting the level of IGF-1. IGF-1 is an important endocrine hormone that regulates growth and promotes the proliferation and osteogenic differentiation of bone marrow mesenchymal stem cells (BMSCs) through the Wnt/β-catenin pathway, which is essential for bone accumulation and maturation (Feng and Meng, 2021). When hepatogenic IGF-1 was regularly increased in aseptic mice, they showed bone loss in the short term but increased bone formation and bone mass after 8 months (Yano et al., 2015). When SCFAs, a metabolite of GM, was supplemented to antibiotic-treated mice, IGF-1 and bone mass were restored to the levels of normal mice, suggesting that the restoration of the GM in germ-free mice is mediated by the production of SCFAs, which induces circulating levels of IGF-1 and thus has an effect on bone metabolism (Yan et al., 2016).

#### 3.3.2 GM affects bone homeostasis through PTH

PTH is a pro-calcium hormone synthesized by the parathyroid glands. Intermittent PTH treatment (iPTH) plays an important role in the regulation of bone development and maturation by stimulating bone formation and resorption. It has been demonstrated (Li et al., 2020) that this effect is dependent on the GM and its metabolites and that iPTH requires physiological concentrations of butyric acid to regulate Treg and induce bone anabolism. At the same time, iPTH requires microbiota to increase the production of TGF-β and IGF-1 in bone marrow. iPTH failed to induce bone formation in germ-free female mice after microbiota removal with broad-spectrum antibiotics.

#### 3.3.3 GM affects bone homeostasis through estrogen

Estrogen plays an important role in bone metabolism, inducing apoptosis in osteoclasts and inhibiting apoptosis in osteoblasts. In an estrogen-deficient environment, the bone conversion cycle is activated more frequently (Ruth et al., 2020). In T-cell-deficient mice, ovariectomy does not lead to bone loss, so TNF plays an important role in bone loss in ovariectomized mice. The key mechanisms by which TNF stimulates bone resorption are activation of RANK and induction of Th17 cells (Yu et al., 2020). IL and TNF inhibition can be used to prevent increased bone resorption due to estrogen deficiency.

In summary, the GM and bone metabolism have a complex and tight association that can affect host metabolism through SCFAs, host immune function through T cells, and host endocrine environment through gut hormones, IGF-1, PTH, and estrogen, thus affecting bone metabolism. As a result, osteoporosis populations have their own specific GM characteristics; that is, diversity and abundance were significantly reduced. Because gut hormones also have a very important influence on bone metabolism, the relationship between the GM, gut hormones, and bone metabolism and their associated mechanisms will be described in detail below.

## 4 GLP-1

GLP-1 is secreted from intestinal L-cells in response to nutrient intake. GLP-1 has two biological activities, namely, GLP-1 (1-36) and GLP-1 (7-36) (Kumar et al., 2016), and GLP-1 (7-36) is predominant in the human body (Ørskov et al., 1994), followed by GLP-1 (7-37) with the same biological activity. Human endogenous GLP-1 cleaves GLP-1 (7-36 amide) and GLP-1 (7-37) on the N-terminal dipeptide very quickly in the presence of dipeptidyl peptidase 4 (DPP-4), generating low-affinity ligands of the GLP-1 receptor, GLP-1 (9-36 amide) or GLP-1 (9-37), which gives it a half-life of only 1.5 min–2 min (Deacon et al., 1995; Kieffer et al., 1995; Mentlein et al., 1993). Subsequently, the kidneys rapidly cleared these intact forms as well as inactivated GLP-1 metabolites. Food composition affects the time and range of GLP-1 secretion. Plasma GLP-1 levels were higher in high-protein diets than in carbohydrate- or fat-rich diets (Lejeune et al., 2006; Raben et al., 2003; Samkani et al., 2018) and increased more slowly after fat than carbohydrate intake (Elliott et al., 1993). Although GLP-1 degradation is not affected by renal function, the elimination of both GLP-1 and its inactive metabolites is prolonged in individuals afflicted by renal insufficiency (Meier et al., 2004).

Evidence has elucidated that GLP-1 orchestrates crucial physiological functions through its interaction with the GLP-1 receptor (GLP-1R), a cAMP-linked G-protein-coupled receptor that is widely distributed throughout the body in various tissues. GLP-1Rs were first identified in pancreatic islet β-cells and the central nervous system (CNS) (Drucker et al., 1987; Shimizu et al., 1987). Subsequently, typical GLP-1R was found to be expressed in the pancreas, kidney, gastrointestinal tract, and blood vessels (Andersen et al., 2018). Recent evidence from Pereira et al. (2015) seems to indicate that the known GLP-1R is also present in bone tissue. GLP-1 acts on pancreatic β to stimulate insulin secretion and improve islet β cell function, on α cells to increase glucose sensitivity and inhibit glucagon secretion, and on δ cells to stimulate growth inhibitory hormone release (Ding et al., 2011; Drucker and Yusta, 2014; Dunning et al., 2005). In addition, activation of GLP-1R in the central nervous system leads to reduced food intake and weight reduction (Holst, 2007), whereas in the stomach, GLP-1 not only inhibits the secretion of gastrointestinal glands but also inhibits gastric motility (especially gastric emptying) and gastric acid secretion (Zhang et al., 2022). *In vivo* tests on healthy volunteers (Schirra et al., 2002) have shown that GLP-1 increases gastric volume, reduces food consumption, and inhibits gastric emptying. In addition to the effects of GLP-1 in healthy individuals, several studies (Deane et al., 2010; Meier et al., 2003; Näslund et al., 1998) have shown that exogenous GLP-1 inhibits gastric emptying in patients with T2DM, obesity, and critical illness. As a result of these beneficial effects of GLP-1R activation on metabolism, several drugs acting on the GLP-1R have been developed for the treatment of T2DM and obesity, including the GLP-1RAs liraglutide and exendin-4 or its synthetic version exenatide, and additional products are in different stages of development (Nauck et al., 2016; Odawara et al., 2016; Oh and Olefsky, 2016; Sheu et al., 2016). Because of the effectiveness of these drugs in the management of metabolic disorders, interest in them has since gradually shifted to bone metabolism.

### 4.1 GLP-1’s effect on bone metabolism *in vitro*

Several previous studies have shown that GLP-1 affects bone metabolism, although the exact mechanisms involved have not been fully explained. GLP-1R has been identified on immature osteoblastic TE-85 (Pacheco-Pantoja et al., 2011), MG-63 (Pacheco-Pantoja et al., 2011), and MC3T3-E1 (Aoyama et al., 2014) cells, whereas it has not been found in the Saos-2 cell line (Pacheco-Pantoja et al., 2011). It has been found that GLP-1R has glucose-dependent expression during osteogenic differentiation of MC3T3-E1 cells induced by bone morphogenetic protein-2 (BMP-2), and its expression increases with the increase of glucose concentration in the culture medium (Aoyama et al., 2014). GLP-1 affects the differentiation and activity of MC3T3-E1 cells by inducing the hydrolysis of glycosylphosphatidylinositol (GPI) and increasing the activities of PI3K and MAPK signaling pathways in these cells, thus promoting cell proliferation, differentiation, and mineralization (Nuche-Berenguer et al., 2010). Furthermore, liraglutide, a GLP-1RA, was shown to directly promote MC3T3-E1 osteogenesis and osteoblast mineralization through the activation of the cAMP/PKA/β-CAT-Ser675 signaling pathway (Wu et al., 2017). It was also proposed that liraglutide regulated MC3T3-E1 cell differentiation mediated by adenosine monophosphate-activated protein kinase (AMPK), a mammalian target of rapamycin (mTOR) signaling (Hu et al., 2016). Moreover, studies by Pal et al. (2019) showed that liraglutide increased the expression of AdipoR1 in osteoblasts in a time-dependent manner. Because the activation of AdipoR1 in osteoblasts leads to the upregulation of the mitochondrial bioproduction factor, Pgc1α, which in turn contributes to their osteogenic effects, it was hypothesized that liraglutide might stimulate mitochondrial function in osteoblasts by upregulating AdipoR1. In addition, liraglutide promotes mitochondrial DNA synthesis in osteoblasts by mediating the upregulation of TFAM. Notably, liraglutide also promotes anti-apoptotic effects in osteoblasts by upregulating the mitochondrial outer membrane protein VDAC1. Therefore, the enhancement of mitochondrial biosynthesis and mitochondrial function seems to play a key role in the effects of liraglutide-induced bone metabolism. Exendin-4, another GLP-1RA, has been shown to enhance the proliferation and differentiation of osteoblasts partly mediated by MAPK pathways, including extracellular signal-regulated kinase1/2 (ERK1/2), p38, and c-Jun N-terminal kinase (JNK) pathways (Feng et al., 2016). The signaling pathway mediated by GLP-1R prevents apoptosis and promotes cell proliferation (Jiang et al., 2016; Wang et al., 2015). In a study by Pacheco-Pantoja and Ranganath (2011), GLP-1 increased cell survival but decreased secretion of P1NPs (markers of bone formation) in two osteoblast lines, MG-63 and TE-85. In another of their studies, it was also found that GLP-1 induced the expression of c-Fos (a gene critical for osteoblast proliferation and differentiation) in osteoblast TE-85 cells (Pacheco-Pantoja et al., 2016). BMSCs have multidirectional differentiation potential and are capable of osteogenic and lipogenic differentiation. It has been shown that GLP-1R is also expressed in BMSCs, and liraglutide promotes osteogenic differentiation, inhibits lipogenic differentiation and reduces cell death, protects against bone loss and fracture, and promotes bone formation in rat and human BMSCs (Jeon et al., 2014; Lu et al., 2015; Meng et al., 2016; Sanz et al., 2010). The effect of GLP-1 on BMSCs has been shown to be mediated in part by mitogen-activated extracellular signal-regulated kinase (MEK) and protein kinase C (PKC) signaling pathways (Sanz et al., 2010). Another study shows that GLP-1 also promotes BMSCs to differentiate into osteoblasts by acting on the PKA/β-catenin and PKA/PI3K/AKT/GSK3β pathways (Meng et al., 2016). The Wnt pathway may mediate GLP-1 to promote bone formation, as exendin-4 treatment has previously been reported to ameliorate bone loss induced by an impaired Wnt pathway in diabetic rats (Nuche-Berenguer et al., 2010). In addition, the present study also observed that exendin-4 decreased the mRNA and protein levels of sclerostin in osteoblast-like MLO-Y4 cells and also decreased the serum sclerostin levels in T2 DM Otsuka Long-Evans Tokushima obese rats. Therefore, it was further inferred that exendin-4 might first bind to GLP-1R and act on the PKA pathway, which in turn affects the Wnt/β-catenin pathway in the osteoblasts, reduces the expression of sclerostin, and promotes the formation of bone (Kim et al., 2013). To ensure the accuracy of the extrapolation, further research is needed before a definitive explanation of this issue can be provided. Moreover, GLP-1R exists not only in osteoblast lines; Li et al. (2020) found that GLP-1R was expressed in mouse BMMS and RAW 264.7 cells and that knockdown of GLP-1R activity increased the expression of pro-osteoclastogenic biomarkers and promoted osteoclast formation and bone resorption. Exendin-4 and liraglutide increased the number of osteoclast precursors in mice, while the addition of mature osteoclasts reduced the absorption area, suggesting that GLP-1RA promoted osteoclast differentiation but inhibited its absorptive activity. The inhibitory effect of liraglutide on osteoclast formation and bone resorption may be achieved by inhibiting the MAPK and NF-κB pathways, which downregulates the expression of downstream osteoclast marker genes mediated by NFATc1 and NFATc1 by GLP-1R. However, whether GLP-1 or GLP-1RA has a direct effect on osteoclast formation is still controversial.

### 4.2 GLP-1’s effect on bone metabolism *in vivo*

Several *in vivo* rodent studies have confirmed that GLP-1 is necessary for bone strength (Yamada et al., 2008). Knockouts (KO) are commonly used in modern medical research. Yamada et al. (2008) showed that tibial and vertebral cortical bone volume and strength were reduced in 10-week-old GLP-1R KO mice compared to wild-type control mice. Although there was no significant change in bone mineral quantity or quality in the GLP-1R KO mice, they had a significantly immature collagen matrix, resulting in reduced cortical layer thickness, bone diameter, bone mineral content, and yield strength (Mabilleau et al., 2013). The trabecular density of GLP-1R and GIPR double KO mice was higher than that of the wild-type control group, but the cortical thickness, cortical area, and external diameter of bone decreased (Mieczkowska et al., 2015). In contrast, treatment with GLP-1RAs has been shown to have osteogenic effects. It has been reported that the treatment of diabetic animal models with exendin-4 can improve bone mechanical properties and prevent bone loss by changing the cortical microstructure and bone composition parameters (mineral crystallinity, collagen maturity, acid phosphate content, and carbon–phosphorus ratio) (Mansur et al., 2019). A study on the role of GLP-1RAs in OVX-induced osteoporosis in rats showed that treatment with exendin-4 for 8 weeks improved the trabecular volume, thickness, and number of lumbar vertebrae and femurs; decreased trabecular spacing; and increased bone mineral density (BMD). This was mainly achieved by increasing the expression of Runx2, alkaline phosphatase (ALP), and Col-1 mRNA. In addition, exendin-4 treatment also increased the expression of p38, p42/44, and β-catenin protein; however, GLP-1RAs had little effect on the mechanical resistance of femurs (Pereira et al., 2015). Another study showed that the exendin-4 also inhibited bone resorption by increasing the OPG/RANKL ratio, thereby preventing deterioration of trabecular microarchitecture, reversing the decline in femur and vertebral bone mass, and increasing bone strength (Nuche-Berenguer et al., 2009). Exendin-4 has been shown to act on the Wnt/β-catenin pathway in osteoblasts via the GLP-1R, thereby promoting bone formation and reducing sclerostin levels in T2DM rats (Kim et al., 2013). Calcitonin’s role in bone metabolism is to inhibit osteoclast activity and attenuate the process of osteolysis. It was found that GLP-1R is expressed in thyroid C cells and promotes calcitonin secretion from these cells via a cAMP-mediated pathway (Crespel et al., 1996; Lamari et al., 1996). GLP-1R KO mice showed higher urinary deoxypyridine (bone resorption markers) levels, decreased cortical bone mass, and increased bone fragility, and bone histomorphometrics showed an increased number of osteoclasts enhanced bone resorption activity (Mabilleau et al., 2013). Urinary deoxypyridine levels were effectively suppressed in GLP-1R KO mice after treatment with calcitonin. These findings confirm the important role of endogenous GLP-1R signals in control, which may be through the calcitonin-dependent pathway (Yamada et al., 2008), but this hypothesis needs to be further explored. GLP-1 may also regulate bone metabolism by affecting blood glucose levels. In particular, hyperglycemia has now been found to be negatively correlated with lumbar spine bone density. It has been shown that GLP-1 controls blood glucose levels by stimulating insulin secretion, inhibiting glucagon secretion, and regulating gastric emptying, thus promoting bone formation (Hare et al., 2009; Sasaki et al., 2015). Therefore, it is speculated that GLP-1 can indirectly affect bone metabolism by regulating the level of blood glucose (Terzi et al., 2015).

In preclinical studies in several rodent models, GLP-1 and GLP-1RA have been shown to have significant benefits on bone metabolism, both of which promote bone formation while inhibiting bone resorption and preventing bone degeneration. However, there is a lack of research on GLP-1 on osteoclasts, and increased research in this area, as well as long-term dynamic monitoring, will help determine the effects of GLP-1 on bone and the specific mechanisms.

### 4.3 GLP-1’s effect on bone metabolism in humans

Compared to rodents, results from human studies are inconsistent. Several researchers (Gilbert et al., 2016; Li et al., 2015) examined BMD and bone turnover markers in T2DM patients treated with liraglutide and exenatide but did not find any effect of GLP-1RAs on bone turnover markers or BMD. However, infusion of GLP-1 reduced CTX-Ⅰ in overweight and obese patients (Bergmann et al., 2019), whereas GLP-1RA exenatide also reduced CTX-Ⅰ levels without affecting the levels of P1NP. In contrast, subcutaneous injection of GLP-1 or its major metabolite, GLP-1 (9–36 amide), did not alter CTX-Ⅰ in overweight (Henriksen et al., 2003) or young healthy adults (Nissen et al., 2019), respectively. While inconsistent with previous results, synthesizing these investigations infers that GLP-1 may inhibit bone resorption while leaving bone formation unaffected in humans. However, weight loss caused by GLP-1RA treatment may mean a higher risk of fracture. A meta-analysis (Mabilleau et al., 2016; Sun et al., 2015) showed that the effects of GLP-1RAs (liraglutide and exendin-4) on fracture risk seem to be inconsistent, with liraglutide significantly decreasing fracture risk, whereas exendin-4 increased fracture risk. Driessen et al. (2015) investigated the incidence of fracture between GLP-1RA users and non-users in the United Kingdom and the Netherlands. The findings demonstrated no significant difference between the two groups, suggesting that the role of GLP-1RAs in a T2DM population is neutral. However, inconsistently, meta-analyses from (Cheng et al., 2019) and (Su et al., 2015) have shown that GLP-1RA treatment has a fracture risk reduction effect in patients with T2DM. Considering the limited number of interventional studies on the effects of GLP-1RA on bone and the lack of sufficient preclinical data, it is reasonable to establish *in vitro* study to investigate the effects of GLP-1 and GLP-1RA on human bone cells, as well as on bone remodeling in individuals with or without diabetes, in order to ensure the accuracy and credibility of the conclusions.

### 4.4 Relationship between GLP-1, GM, and bone metabolism

The role of GLP-1 in improving bone metabolism and anti-osteoporosis has been discussed above, although the studies on the promotion of GLP-1 secretion by SCFAs are not yet in-depth, and the related mechanisms are not yet clear. GLP-1, as a gut hormone, may be closely related to the GM and bone metabolism to a large extent (Han et al., 2021; Wang et al., 2020). Here, a previous study showed that consumption of the probiotic VSL 3 promoted GLP-1 secretion in mice, accompanied by increased fecal butyric acid levels (Yadav et al., 2013). Butyric acid has also been shown to stimulate the proliferation of intestinal mucosal cells, expanding the surface area of the intestinal epithelium and further enhancing calcium absorption, suggesting an inextricable link (Chen et al., 2019). In contrast, previous studies have shown that some GMs produce serotonin that crosses the intestinal barrier and enters the bloodstream and that serotonin reduces the secretion of GLP-1, which in turn reduces the production of osteoblasts, inhibits bone formation, and leads to osteoporosis (Yadav et al., 2010).

**FIGURE 3:**
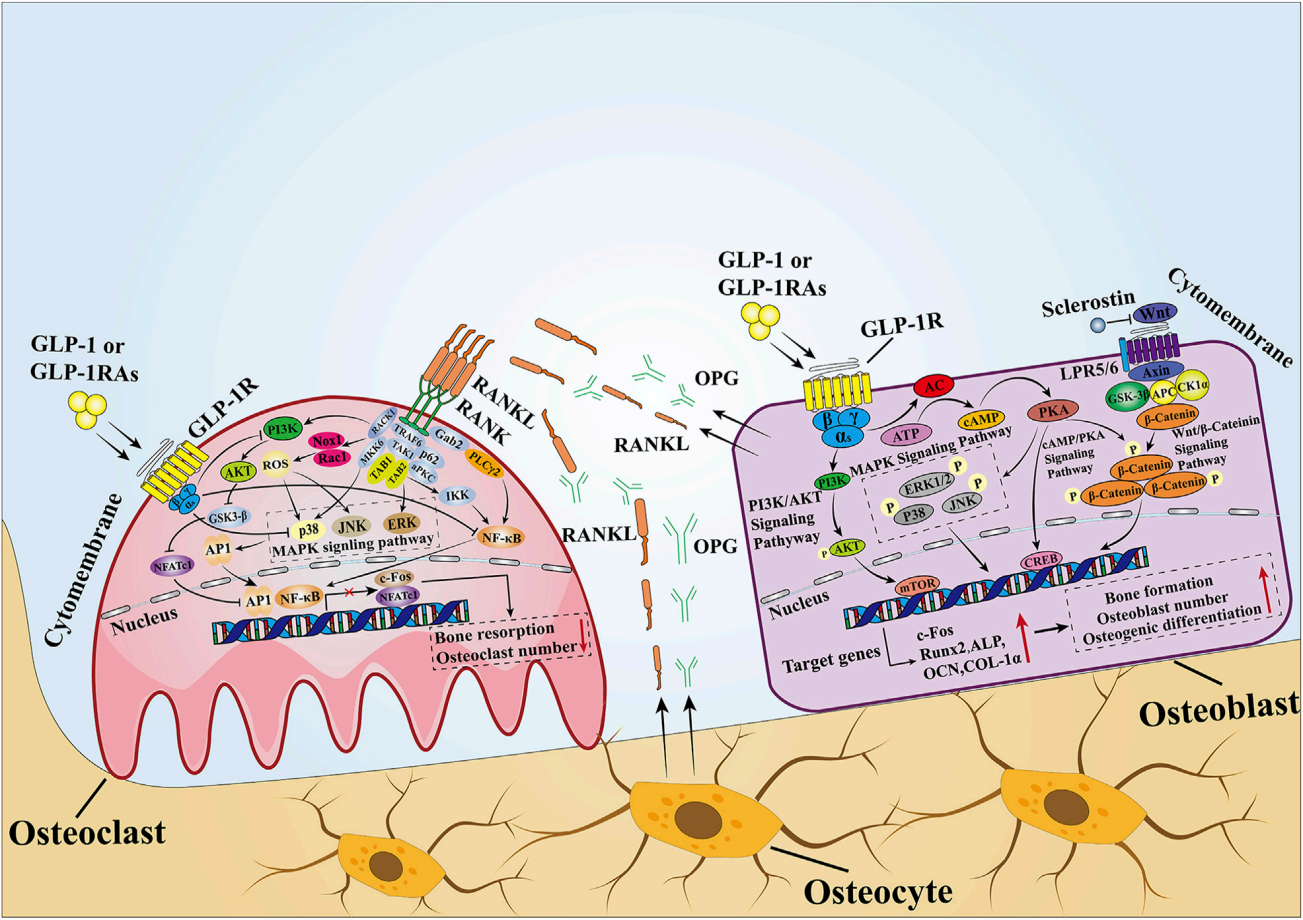
Mechanisms of GLP-1 effects on bone. In MSCs and osteoblasts, GLP-1 activates intracellular signaling pathways by binding to its receptors. After GLP-1R is activated, it stimulates adenylate cyclase to convert ATP into cAMP and then activates downstream PKA signals. Then, PKA continues to increase the phosphorylation of ERK, P38, JNK, and β-catenin, causing them to enter the nucleus to stimulate the expression of osteogenesis-related genes and promote bone formation. In osteoclasts, when GLP-1 binds to GLP-1R, it inhibits the activity of the intracellular MAPK pathway and NF-κB pathway. The former downregulates the transcription of c-Fos to inhibit osteoclast formation, while the latter downregulates the expression of downstream osteoclast marker genes mediated by NFATc1 and NFATc1 to inhibit bone resorption. In addition, GLP-1 directly inhibits the activity of RANKL, thus inhibiting the activity of the NF-κB pathway. OPG and RANKL secreted by osteoblasts and osteoclasts are important signaling pathways that mediate the balance between bone resorption and bone formation, and a proper state of balance is conducive to maintaining bone homeostasis.

In summary, many studies have shown that GLP-1 affects bone mass and bone quality by affecting bone formation and bone resorption. Although the molecular pathways between GLP-1 and bone metabolism are summarized in this review (Figure 3), the specific processes of GLP-1 or GLP-1RAs affecting bone metabolism and their related molecular mechanisms have not been fully elucidated in different parts of bone tissues from patients with different metabolic states. Therefore, further studies on the effects of GLP-1 or GLP-1RAs on bone metabolism under different metabolic states are necessary. Finally, because GLP-1 is a good metabolic regulator hormone, elucidating the specific mechanism of the effect of GLP-1 on bone metabolism will help guide the development of new drugs for osteoporosis.

## 5 GLP-2

Like GLP-1, GLP-2 is secreted by L-cells in the small and large intestine after nutritional intake, and GLP-2 secretion was not as high after ingestion of a carbohydrate-rich meal as it was after ingestion of protein and fat (Raben et al., 2003). GLP-2 is a short peptide of 33 amino acids derived from the same prepeptide-proglucagon that is decomposed to produce GLP-2 under the action of pre-hormone converting enzyme 1/3 (PC1/3). There are two main forms of GLP-2 in the human body, which are bioactive GLP-2 (1-33) and non-bioactive GLP-2 (3-33) (Hansen et al., 2007). The basal level of plasma GLP-2 is very low during fasting. The concentration of GLP-2 will increase rapidly after eating, and then it will be hydrolyzed by DPP-4 at the first two amino acid residues of GLP-2 (1-33) to non-bioactive GLP-2 (3-33). Although the half-life of GLP-2 (3-33) is about 7 min (Hartmann et al., 2000), it is much longer than that of GLP-1 (1.5 min–2 min). There are two ways to extend the half-life of GLP-2: using DPP-4 inhibitors or substituting 2-position alanine (Hartmann et al., 2000; Jeppesen et al., 2005; Thulesen et al., 2000).

According to the study, most of the physiological effects exerted by GLP-2 are mediated by the GLP-2 receptor (GLP-2R). The GLP-2R belongs to class B1 of the G-protein-coupled receptors (GPCRs) and is highly specific for GLP-2, based on rodent studies. The GLP-2R is widely expressed throughout the intestine but is also found in the central nervous system and possibly to a lesser extent in the lungs (Yusta et al., 2000). In humans, however, the distribution of GLP-2R remains uncertain due to the lack of well-immunolocalized antibodies and the low expression of GLP-2R in cells outside the gastrointestinal tract. Extrapolation from other species may be risky due to interspecies variation (Drucker and Yusta, 2014).

GLP-2 promotes intestinal nutrient absorption. In mice, GLP-2 promoted the growth of the small intestine and large intestine, and inconsistently, the combination of GLP-2 and high-dose GLP-2 (3-33) reduces the growth-promoting effect, speculating that it may be due to competitive antagonism between GLP-2 and GLP-2 (3-33) (Thulesen et al., 2002). GLP-2 acts on intestinal crypts to stimulate cell proliferation and inhibit apoptosis (Dubé et al., 2006). Although less well-established, it possesses the capacity to suppress food intake and bolster the growth of neurons (Askov-Hansen et al., 2013; Bremholm et al., 2010; Henriksen et al., 2009). As a result, a DPP-4-resistant GLP-2 analog (teduglutide) has been used in the treatment of short bowel syndrome (SBS) since 2012 (Jeppesen et al., 2018). GLP-2 promotes the absorption of intestinal nutrients and blood supply. Studies have shown that GLP-2 can increase the blood supply of the superior mesenteric artery through vasoactive intestinal peptide (VIP) and 5-hydroxytryptamine (5-HT) (Guan et al., 2006). GLP-2 inhibits gastric acid secretion. Sham-feeding increased gastric acid secretion, and this increase was reduced by 65% when GLP-2 was given compared to saline, suggesting that GLP-2 can inhibit gastric acid secretion in humans (Meier et al., 2006). Moreover, as GLP-2 regulates glucose metabolism, endogenous GLP-2 can be used as a protective factor against glucose metabolism disorders in mice fed with a high-fat diet, and chronic treatment of GLP-2 (3-33) improves glucose metabolism disorders (Baldassano et al., 2015). The significant role of GLP-2 in participating in the body’s metabolism raises the question: does GLP-2 play a role in bone metabolism?

### 5.1 GLP-2’s effect on bone metabolism *in vitro*

Similar to the GLP-1, GLP-2 may have practical value in the treatment of osteoporosis. However, the mechanisms by which GLP-2 acts on bone are not fully understood. Pacheco-Pantoja et al. (2011) discovered the expression of GLP-2R in the immature human osteoblast cell lines TE-85 and MG-63. After incubating GLP-2 with serum-deficient osteoblast cell line TE-85, the level of P1NP decreased, indicating that bone formation may be reduced. However, another parameter associated with bone formation (ALP levels) was unchanged (Pacheco-Pantoja et al., 2011). In contrast, neither P1NP nor ALP changed in the MG-63 osteoblast line, but osteocalcin (OCN) decreased after 5 days of GLP-2 treatment (Pacheco-Pantoja et al., 2011). No significant response was observed in the most mature cell line, Saos-2, which is not surprising because we did not detect the presence of GLP-2R mRNA in the Saos-2 cell line (Pacheco-Pantoja et al., 2011). In addition, recent reports have also indicated that GLP-2R was not detected in human MSC expression (Jeppesen et al., 2018). There seems to be some inconsistency regarding the response triggered by GLP-2, as some bone formation markers were changed while others were unaffected. By contrast, GLP-2R is expressed in human osteoclasts (Skov-Jeppesen et al., 2019), suggesting that GLP-2 may regulate the activity of osteoblasts and possibly that of osteoclasts through coupling factors secreted by osteoclasts. Therefore, it is necessary to investigate the effects of GLP-2 on osteoclasts and osteoblasts and their mechanisms.

### 5.2 GLP-2’s effect on bone metabolism *in vivo*

*In vivo* experiments show a more divergent role and application of GLP-2 in bone mass. The preliminary results of GLP-2 in SBS rats showed that the BMD in the spinal region increased significantly after 5 weeks of GLP-2 treatment (Scott et al., 1998). Huang et al. (2023) showed that GLP-2 treatment for 6 weeks could reduce bone loss in SAMP6 mice, which was characterized by increased bone mineral density, improved microstructure of the femur, and enhanced osteogenic activity. However, the activity of osteoclasts was not significantly inhibited. Further exploration revealed that GLP-2 decreased the level of TNF-α, increased the expression of intestinal GLP-2R and ileal vitamin D receptor, and improved intestinal oxidative stress by increasing GPX-4 and SOD-2 signaling pathway. In contrast, Gobron et al. (2020) showed that GLP-2 can not only promote the expression of bone matrix genes and promote collagen maturation, but it also reduces the number of newly formed osteoclasts in a dose-dependent manner *in vitro*. However, GLP-2 can neither improve the bone strength of the femoral shaft or lumbar vertebrae nor improve the bone microstructure, and it does not provide a real beneficial effect in improving bone strength in the brittle-bone mouse model.

### 5.3 GLP-2’s effect on bone metabolism in humans

In contrast to rodent research, various human studies have recognized the superior bone-promoting properties of GLP-2. It was found that treatment with natural GLP-2 for 5 weeks significantly increased the BMD in SBS patients without a terminal ileum and colon (Haderslev et al., 2002; Jeppesen et al., 2001). Henriksen et al. (2003) showed that in healthy postmenopausal women, subcutaneous injections of GLP-2 (dose range 200 μg–800 μg) were able to reduce bone resorption (as measured by CTX-I) in a dose-dependent manner, with no significant effect on bone formation (as measured by BMD). In addition, it was found that GLP-2 was effective in inhibiting nocturnal bone resorption (measured by CTX-I) after injection at bedtime (Henriksen et al., 2004). To confirm these findings, a longer 14-day study was conducted, which revealed that markers of bone resorption regulated by enteroendocrine hormones and bone physiology were indeed significantly reduced, whereas indicators of bone formation remained unchanged (Henriksen et al., 2007). Another study with a longer period showed that 4 months of GLP-2 treatment resulted in a significant and sustained reduction in bone resorption and an increase in femoral neck and total hip bone density, as assessed by biomarkers, in 160 postmenopausal women with bone loss. (Henriksen et al., 2009). Consistent with the preceding discoveries, which indicated that GLP-2 enhanced a BMD in individuals with SBS (Haderslev et al., 2002), the research conducted by Gottschalck et al. (2008) showed that the diminishment of CTX-Ⅰ subsequent to the administration of exogenous GLP-2 necessitates an intact small intestine, thus implying an indirect influence of GLP-2 on the intestinal system. Interestingly, teduglutide has been shown to increase whole-body bone mineral content (BMC) in SBS patients, although there was no increase in lumbar spine or hip bone density, suggesting that the drug may have a positive effect on bone-like GLP-2 (Jeppesen et al., 2011). While the mechanism of GLP-2’s effect on bone mass remains unclear, it can be speculated from a study by Haderslev et al. (2002) that GLP-2 may increase bone matrix mineralization by promoting increased intestinal calcium absorption. Delightfully, Gottschalck et al. (2008) discovered a fascinating correlation, whereby GLP-2 exhibited a reduction in PTH levels among the control individuals possessing an intact intestinal system, thus establishing PTH as a plausible mediator of the GLP-2-induced decline in CTX-Ⅰ. In patients with hypoparathyroidism (due to thyroidectomy), the role of exogenous GLP-2 is absent (Skov-Jeppesen et al., 2021). Surprisingly, despite the impact exerted by GLP-2 on osteoclastic function, the presence of GLP-2R remains elusive within human osteoblasts or any other analogous cell lineage associated with skeletal tissue, although Pacheco-Pantoja et al. (2011) found that this receptor is expressed in the immature human osteoblast cell lines MG-63 and TE-85. This led to the hypothesis that GLP-2 either acts directly on osteoclasts (rather than osteoblasts) or operates via secondary signaling factors.

### 5.4 Relationship between GLP-2, GM, and bone metabolism

GLP-2 improves bone metabolism abnormalities caused by osteoporosis (He et al., 2019; Lu et al., 2018; Wu et al., 2018). In a study on aged rats, it was found that aging caused a decrease in the expression of gut-tight junction proteins, an increase in intestinal permeability, and an enhancement of chronic inflammation, which led to an increase in the levels of circulating inflammatory factors, such as IL-1 and IL-6, and the activation of the RANK/RANKL/OCN signaling pathway, which promoted the proliferation of osteoclasts and enhanced bone resorption (Ren et al., 2014). In an experimental study in ovariectomized rats, GLP-2 inhibited circulatory inflammation, improved the microstructure of trabecular bone, inhibited bone resorption, and promoted bone formation (He et al., 2019). In addition, it has been reported that GLP-2 can regulate the GM of aged rats, and the abundance of the Spirochete phylum was significantly reduced in GLP-2-intervened aged rats compared with the control group. Further studies are needed to investigate whether GLP-2 can improve osteoporosis by improving the GM (Wu et al., 2018).

In summary, combining the results of preclinical and clinical studies, GLP-2 has been proven to play a significant role in inhibiting bone resorption but has little effect on bone formation, thus promoting the increase of BMD. Current studies have shown that only supraphysiological doses of GLP-2 can effectively reduce bone resorption (CTX-I), but the specific mechanism of GLP-2 on bone metabolism is not yet fully articulated. It may be through direct action on osteoclasts, or it may involve the gut axis or other neurological factors; the exact mechanism is worth further discussion.

## 6 GIP

GIP, a peptide consisting of 42 amino acids, is predominantly synthesized and released by the K-cells found in the duodenum and jejunum of the small intestine in response to the ingestion of food. In conjunction with GLP-1, GIP is classified as an incretin hormone as it plays a crucial role in regulating glucose metabolism. Remarkably, GIP has been shown to account for about half of the insulinotropic effect upon oral glucose administration in healthy individuals (Baggio and Drucker, 2007). Consumption of diets rich in carbohydrates or fats results in higher GIP levels than high-protein diets in both healthy and T2DM patients (Elliott et al., 1993; Park et al., 2015), and GIP levels rise higher and more rapidly after fat intake than carbohydrate or protein intake (Raben et al., 2003). GIP is derived from pro-peptide (Pro-GIP), a 153-amino acid precursor hormone expressed in intestinal endocrine K-cells, pancreatic α cells, and possibly expressed in central nervous system cells in the upper intestine (Finan et al., 2016), which is then cleaved by post-translational processing into a biologically active form of GIP (1-42) (Drucker, 2006). GIP (1-42) is composed of 42 amino acids, mainly encoded by exons 3 and 4, and is released from its precursor by post-translational cleavage dependent on hormone-converting enzyme PC1/3 (PC1/3). It is located on both sides of a single arginine residue (Ugleholdt et al., 2006), but there is also a naturally occurring C-terminally truncated variant, devoid of the last 12 amino acids, GIP(1-30)NH2, which acts as full agonist for the human GIP system (Hansen et al., 2016) and is produced in intestinal and pancreatic α-cells by PC2 cleavage of Pro-GIP (Finan et al., 2016). Both forms have the same insulin-promoting effect in mice (Gault et al., 2011). Similar to GLP-1, GIP is rapidly degraded by DDP-4 to the metabolite GIP (3-42); the active GIP has a half-life of only 4 min in plasma and is quickly removed from circulation through the kidney (Vilsbøll et al., 2006). Therefore, the cycle level of complete bioactive GIP is very low. In contrast, GIP(1-30)NH2 is cleaved by DPP-4 to produce GIP(3-30)NH2, a high-affinity competitive antagonist of the GIP system that has been shown to be active in humans (Asmar et al., 2017; Gasbjerg et al., 2018; Hansen et al., 2016). For research purposes, multiple GIP analogs that resist DPP-4 degradation have been synthesized, including N-AcGIP (Mabilleau et al., 2014), Pro^3^GIP (Yang et al., 2022), and D-Ala^2^-GIP (Killion et al., 2020) to study various metabolic diseases.

GIP plays its physiological role by binding to the GIP receptor (GIPR), a B7-transmembrane G-protein-coupled receptor related to the GIP-, GLP-2-, and glucagon receptors and, like these, belongs to the glucagon receptor family (Finan et al., 2016) and mainly couples to Gαs. It is widely expressed throughout the body (e.g., pancreas (Moens et al., 1996), adipose tissue (Yip et al., 1998) and bone (Bollag et al., 2000)) and exerts certain biological activities. Accordingly, the signaling of GIPR has been demonstrated in pancreatic α- and β-cells (Moens et al., 1996), bone cells (Bollag et al., 2000), and adipocytes (Yip et al., 1998). Compared with the GLP-1 system (Gabe et al., 2018; Sparre-Ulrich et al., 2017), the GIP system is less conservative among species; as a result, the sequence homology of rodent and human GIPR is only 81%, and the effect of human GIP on rat and mouse GIPRs is only 75% and 60% of rat and mouse GIP, respectively. GIP is best known for its ability to act on islets, where it regulates blood glucose levels by stimulating the secretion of insulin or glucagon (Christensen et al., 2011) and exerts its hypoglycemic effect by stimulating the Gα subunit, which in turn activates the adenosine–adenylate cyclase (AC)–cAMP-PKA signaling pathway when GIP binds to its receptor. A study found that long-term overexpression of GIP in mice not only improved glucose homeostasis but also reduced diet-induced obesity and steatosis (Kim et al., 2012). In contrast, GIPR KO mice are protected from the development of high-fat diet-induced obesity and insulin resistance (Miyawaki et al., 2002). Due to the evident decline in the insulinotropic impact of GIP on individuals with T2DM (potentially attributed to the desensitization of the GIP system) (Vilsbøll et al., 2003), the focus of GIP research has shifted from glucose homeostasis to other areas, such as bone metabolism and other diseases.

### 6.1 GIP’s effect on bone metabolism *in vitro*

In the past decade, GIP has been deemed a vital catalyst for bone remodeling, playing a crucial role in the optimization of bone quality. GIP functions in a dualistic fashion, exerting its influence on bone quality through both pro-anabolic and anti-catabolic mechanisms. Compared to GLP-1R, GIPR was observed to be present in cell lines derived from both osteoblasts and osteoclasts (Pacheco-Pantoja et al., 2011). Additionally, GIPR expression was detected in primary cultures of murine osteoclasts and osteoblasts (Ding et al., 2008; Zhong et al., 2006). The expression of the GIPR has also been confirmed on hBMSC (Berlier et al., 2015). GIP has been shown to regulate osteoblast activity by increasing bone formation parameters such as ALP (Bollag et al., 2000), P1NP (Pacheco-Pantoja et al., 2011), and intracellular calcium [Ca^2+^]_i_ in human osteoblast-like cell cultures. Exposing three human osteosarcoma cell lines expressing GIPRs (TE-85, MG-63, and Saos-2) to GIP resulted in the release of bone formation marker P1NP (Pacheco-Pantoja et al., 2011), in which the expression of Col-1 in Saos-2 cells increased (Bollag et al., 2000). MC3T3-E1 cells exposed to GIP can increase cAMP levels, promote collagen maturation, and regulate the diameter of collagen fibers (Mieczkowska et al., 2015). Furthermore, GIP also increases the expression of c-Fos, a crucial factor in bone cell proliferation and differentiation (Pacheco-Pantoja et al., 2016). Another role of GIP appears to be safeguarding cell integrity. Specifically in osteoblasts and hBMSCs, GIP mitigates the pervasive apoptosis observed amid serum deprivation in tissue culture by impeding the activation of caspases 3/7, hence shielding against cellular harm (Berlier et al., 2015). GIP acts on both osteoblasts and osteoclasts. After GIP treatment with osteoclast-osteoblast co-culture, bone resorption decreased, and osteoblast survival rate increased. The effects of GIP on osteoclasts and osteoblasts were eliminated by GIP(3-30)NH2, a GIP receptor antagonist (Hansen et al., 2023). GIP restrains PTH-induced bone resorption in primary cultured mouse osteoclasts (Zhong et al., 2006) and diminishes the elevated intracellular calcium concentration ([Ca^2+^]i) and calcineurin activity induced by RANKL. In addition, it attenuates the nuclear translocation of NFATc1, a crucial downstream effector of the RANKL signaling pathway that is indispensable for the terminal stage of osteoclast differentiation (Mabilleau et al., 2016). Further investigation shows that a GIP analog diminished the differentiation and bone resorptive efficacy of human osteoclasts (Mabilleau et al., 2016), and treatment of human osteoclasts with GIP analogs resulted in a diminution of gene expression pertaining to distinctive markers of osteoclasts as well as a decline in the quantity of nascent osteoclasts (Gobron et al., 2020), suggesting a direct influence of GIP on human osteoclasts.

### 6.2 GIP’s effect on bone metabolism *in vivo*

Numerous mouse models have been used to assess the effects of GIP on bone, including GIP injections, GIPR KO mice, GIPR overexpressing mice, and the use of DPP-4 resistant peptides. In 2001, the first *in vivo* research conducted by Bollag et al. (2001) showed that native GIP positively affects BMD in OVX rats, and intravenous injection of GIP restored the BMD of the spine (evaluated by dual-energy X-ray absorption scanning), which returned to the same level as the unovariectomized control group 6 weeks later. The DPP-4 resistant peptides showed anabolism or anti-absorption characteristics; for example, N-AcGIP exerted a beneficial impact on the properties of cortical bone in rats and attenuated osteoclast-induced bone resorption in OVX mice, as evidenced by a reduction in osteoclast abundance and the absorption marker CTX-Ⅰ (Mabilleau et al., 2014; Mabilleau et al., 2016). In a mouse model of T1DM, short-term treatment with D-Ala^2^-GIP prevented a decrease in bone formation parameters (collagen maturation index) and improved mechanical properties at the tissue level (Mansur et al., 2015).

Two variants of GIPR KO mice exist, differing in the extent of exonic deletions. Both models of GIPR KO mice lead to inactivity of the GIPR pathway, but certain findings are conflicting. In GIPR KO mice lacking exons 4-5 of the GIP receptor gene, it was observed that bone size and BMD decreased, trabecular morphology and volume changed significantly (Tsukiyama et al., 2006; Xie et al., 2005), and bone formation parameters such as ALP, OCN, and trabecular volume decreased, whereas resorption parameters such as osteoclast number and urinary excretion of the resorption marker deoxypyridin increased, indicating that GIP promoted bone formation and inhibited bone resorption and thus had a comprehensive anabolism effect on bone mass (Xie et al., 2005; Tsukiyama et al., 2006). Apart from this, the postprandial serum calcium levels of these mice were higher, suggesting that GIP may play a role in skeletal calcium deposition (Tsukiyama et al., 2006). In contrast, in GIPR KO mice lacking exons 1-6 of the GIP receptor gene, mechanical resistance and cortical thickness decreased due to increased bone resorption, and osteoclast numbers were reduced despite an increase in osteoblast numbers (Gaudin-Audrain et al., 2013; Mieczkowska et al., 2013). To further confirm whether the bone phenotype observed in GIPR KO mice is directly deficient in GIPR or the compensatory mechanism caused by increased sensitivity to GLP-1, Mieczkowska et al. (2015) evaluated the bone phenotype of dual insulin receptor (GLP-1R and GIPR) KO mice (DIRKO) and found a reduction in the cortical properties and a reduced of bone strength in this mouse, which is consistent with the crucial function of GIP as a gastrointestinal hormone linking nutrient intake and bone formation. This clearly demonstrates the osteoprotective effects of GIP, but the effects of GIP may be indirectly stimulated through extraosseous GIPR activation (Mabilleau et al., 2018). However, a study by Baldassano et al. (2019) found that 3 weeks after injection of specific GIPR antagonists (GIP (3-30)NH2) or specific GLP-2R antagonists (GLP-2 (11-33) and GLP-2 (3-33)), neither GIPR antagonists nor GLP-2R antagonists affected bone resorption in rats, reflecting interspecies differences. Congenital GIP deficiency shows a vital role in bone metabolism, similar to GIPR KO, which is characterized by a notable reduction in bone volume and trabeculae accompanied by an escalation in the osteoclast population (Nasteska et al., 2014). In contrast, administration or overexpression of GIP is linked to augmented osteogenesis, evidenced by elevated bone density, heightened osteoblast tally, elevated OCN concentrations, and suppression of bone resorption, as denoted by reduced pyridinoline crosslinks and diminished osteoclast populace. In transgenic mice whose systemic GIP levels were two to three times higher than those of wild-type mice, the BMD increased by about 5%, while the bone formation marker OCN and the number of osteoblasts increased, but bone resorption, characterized by a decrease in the number of osteoclasts, was inhibited (Ding et al., 2008; Xie et al., 2007).

In a word, investigations from rodent studies indicate that GIP possesses antiresorptive properties on bone and potentially enhances bone formation, thereby implying a linkage between GIP and bone metabolism. It is imperative to conduct further investigations elucidating the underlying mechanisms through which GIP impacts bone cell activity and the interplay between bone formation and resorption.

### 6.3 GIP’s effect on bone metabolism in humans

Postprandial inhibition of bone decomposition has been well-documented in human subjects (Clowes et al., 2002). Although Henriksen et al. (2003) found no significant effect of brief intravenous GIP on bone resorption, multiple investigations have found that GIP infusion inhibited the bone resorption marker CTX-Ⅰ (Bergmann et al., 2019; Christensen et al., 2018; Nissen et al., 2014; Skov-Jeppesen et al., 2021). The contribution of endogenous GIP has been reported to be as high as 25% of the observed reduction in postprandial bone resorption in humans (Helsted et al., 2020). Infusion of GIP in healthy and T1DM patients reduced CTX-Ⅰ but not P1NP, and these effects of GIP on bone were inhibited by selective GIPR antagonists (Nissen et al., 2014; Westberg-Rasmussen et al., 2017; Christensen et al., 2018; Skov-Jeppesen et al., 2019). More recently, emerging research has revealed that GIP exhibits the capacity to attenuate CTX-Ⅰ levels while concurrently augmenting OCN and P1NP concentrations in juveniles, suggesting that GIP might actively contribute to bone anabolism in addition to its inhibitory effects on bone resorption (Skov-Jeppesen et al., 2019). Glucose intake and infusion of GIP decrease PTH levels (Polymeris et al., 2011). However, the antiresorptive effect of GIP is preserved in overweight or hypoparathyroidism individuals (Bergmann et al., 2019; Skov-Jeppesen et al., 2021), suggesting that GIP affects bone metabolism independently of PTH. Regardless of the effects on bone formation, it seems that administration of GIP induces a swift decoupling of bone remodeling in humans, which, if sustained, could potentially result in augmented bone density. While these investigations propose acute, antiresorptive, and conceivably anabolic consequences of GIP in humans, the long-term influence of GIP treatment on bone remodeling, enhancement of bone integrity, and augmentation of BMD await validation through clinical trials. Interestingly, the combined application of GLP-1 and GIP resulted in a greater decrease in bone resorption than was seen with these hormones alone (Bergmann et al., 2019), suggesting that GLP-1/GIPR dual agonists, currently used as therapeutic agents for T2DM, may have significant antiresorptive effects (Frias et al., 2018). Thus, although GLP-1R/GIPR agonists may result in significant weight loss, the co-administration of dual GLP-1R/GIPR agonists could potentially shield patients from concurrent osteoporosis by exploiting the inherent antiresorptive properties of GIP and GLP-1.

Recently, in light of the advent of DPP-IV inhibitors, which serve as enzyme inhibitors for insulin hormone degradation and are being used as therapeutic interventions for individuals with diabetes, there has been a mounting curiosity surrounding the impact of these pharmaceutical agents on skeletal well-being (Meier et al., 2016). In an investigation conducted on human diabetic patients (Bunck et al., 2012), patients were randomly divided into control groups or treated with DPP-IV inhibitor vildagliptin for 1 year. There was no difference in markers for bone fracture (sCTX) or bone formation (ALP) among the groups. Likewise, within a meta-analysis encompassing 22,961 individuals afflicted with diabetes, the utilization of DPP-IV inhibitors exhibited no discernible linkage with a diminishment in fractures (Driessen et al., 2017). The same group performed a retrospective investigation encompassing 328,254 individuals who were prescribed antidiabetic medications as per the Clinical Practice Research Datalink (CPRD) database (Driessen et al., 2017). The authors discovered no discernible linkage between the prolonged administration of DPP-IV inhibitors (spanning from 4 to 8.5 years) and the propensity for osteoporosis or hip fracture. Consequently, the collective accord derived from these constrained investigations thus far appears to intimate that the utilization of DPP-IV inhibitors in the management of individuals afflicted by diabetes mellitus does not engender a depreciation in bone density nor augment the likelihood of fracturing. Nevertheless, it is unclear whether these drugs help increase bone mass or bone quality in normal or diabetic patients. A functional polymorphism of the GIPR affects bone metabolism. A study of 1,686 women conducted by (Torekov et al., 2014) showed that compared with non-carriers, women with Glu354Gln (Rs1800438), a common variant of GIPR, had lower expression of human islet GIPR mRNA and lower cortical BMD in the hip, but no significant difference in lumbar vertebrae. Their risk of non-spinal fracture increased by more than 50%. Moreover, the presence of a deleterious GIPR gene variant (E354Q) was associated with reduced BMD, as assessed through DXA scans in a longitudinal study spanning a decade and involving 1,424 perimenopausal females, and an assessment of fractures registered over a 16-year period showed a 50% increase in fracture risk (Torekov et al., 2014).

In conclusion, preclinical studies have shown that GIP exerts a pivotal and direct influence on the intricate regulation of bone homeostasis. GIP improves bone quality in two ways: it can promote anabolism, and it can resist catabolism. This dual effect is critical for the regulation of bone metabolism, although its exact mechanism remains to be elucidated.

## 7 PYY

PYY is synthesized and secreted by pancreatic endocrine cells (PP cells) and distal open intestinal endocrine cells located in the small intestine and colon in response to nutrient intake. PYY is usually co-secreted with GLP-1 and GLP-2 (Adrian et al., 1985), proportional to calorie intake, and reduces food intake via appetite suppression involving the hypothalamic arcuate nucleus. Compared to GIP and GLP-1, the secretion of PYY seems to be independent of the composition of the macro-nutrients consumed (Samkani et al., 2018). Two endogenous forms of PYY exist: the 36-amino acid form, PYY(1-36), and the 34-amino acid, NH2-terminally truncated form, PYY(3-36) (Medeiros and Turner, 1994; Mentlein et al., 1993; Savage et al., 1987). PYY(3-36) is produced by DPP-4 cleavage of tyrosine–proline residues at the end of PYY(1-36) NH2 in capillary endothelial, liver, and blood cells (Ballantyne, 2006; Grandt et al., 1994; Medeiros and Turner, 1994). Because it is extremely difficult to determine PYY(3-36), the plasma level described here refers to total PYY (Toräng et al., 2016). Plasma PYY levels typically begin to rise within 15 min–30 min after a meal, reach the maximum at 60 min–90 min after a meal, and last for several hours (Steinert et al., 2017).

In general, different forms of PYY molecules have different half-lives and function through different G-protein-coupled Y receptors, and they have different affinities to these receptors. PYY (1-36) acts by activating a variety of neuropeptide Y family receptors, including NPY1R (or Y1R), NPY2R (or Y2R), NPY4R (or Y4R), and NPY5R (or Y5R), while PYY (3-36) is highly selective for NPY2R (or Y2R) expressed throughout the body (Abounader et al., 1995; Simpson et al., 2012; Walther et al., 2011). NPY1R (Larhammar and Salaneck, 2004) and NPY2R (Shi et al., 2010) exhibit ubiquitous expression across various bodily regions, encompassing numerous cerebral regions, the gastrointestinal tract, and vagal afferents. In addition, NPY2R (Nguyen et al., 2011) is expressed mainly in the human ileum, colon, pancreatic gland, and prostate regions, whereas NPY4R (Lin et al., 2009) and NPY5R (Reichmann et al., 2016) are mainly expressed in brain tissue. Both PYY(1-36) and PYY(3-36) suppress appetite and food intake, delay gastric emptying, and regulate pancreatic β-cell survival through appetite suppression involving the arcuate nucleus of the hypothalamus (Moran et al., 2005; Sam et al., 2012). Intravenous injection of PYY (3-36) in rats was 10 times more potent in suppressing feeding than an injection of PYY (1-36) and 4–8 times more potent in suppressing feeding than an intravenous injection of PYY(3-36) in humans (Steinert et al., 2017). However, it was surprising that central injection of PYY (1-36) stimulated food intake in rats (Steinert et al., 2017); the reason is unclear.

Consistent with the positive effects of satiety, PYY levels were found to be heightened among individuals suffering from anorexia and diminished in those afflicted with obesity. In obese participants, diet-induced PYY secretion decreased, but the anorexia effect of PYY seemed to be intact (Batterham et al., 2003). The postprandial circulating levels of PYY are significantly elevated subsequent to undergoing bariatric surgery, especially after interventions that expedite the swift distribution of nutrients to the L-cells located in the ileum and colon, as is the case with Roux-en-Y gastric bypass procedures (Yang et al., 2019). The heightened PYY levels observed after bariatric surgery have the potential to diminish appetite and subsequent food consumption. Elevated PYY levels after bariatric surgery may lead to decreased appetite and food intake, as exogenous administration of PYY (3-36) analogs leads to decreased food intake and weight loss in rodents and human clinical trials (Chandarana et al., 2011; Nishizawa et al., 2017; Tan et al., 2017). Therefore, protracted PYY (3-36) analogs reduce body weight in rodents, and PYY analogs are currently being investigated for the treatment of obesity (Leitch et al., 2019). More significantly, these drugs may exert a profound effect on bone metabolism.

### 7.1 PYY’s effect on bone metabolism *in vivo*

Evidence from rodent studies supports that PYY regulates bone homeostasis by modulating osteoclast and osteoblast activity; in addition, PYY can indirectly regulate bone homeostasis through interactions with the hypothalamus. Even though PYY interacts with various Y receptors, it is noteworthy that only the Y1R is expressed in mouse BMSCs and osteoblasts, and PYY may exert an inhibitory effect on osteoblast activity through these receptors (Lee et al., 2011; Lundberg et al., 2007). Accordingly, an excessive production of PYY in genetically modified mouse models resulted in a decline in bone density, whereas the absence of Y1R elevates the expression of osteogenic transcription factors, promotes the formation of mineralized nodules *in vitro*, and augments the functionality of osteoblasts on both the intracortical and periosteal surfaces. Consequently, these effects contribute to the development of larger skeletal structures and increased trabecular bone volume (Lee et al., 2010; Lee et al., 2011; Wong et al., 2012). While a distinct murine KO model of the PYY gene, generated using different genetic backgrounds, exhibited reduced trabecular mass and strength alongside increased bone loss following ovariectomy (Wortley et al., 2007), the majority of existing evidence supports the notion of an antagonistic influence exerted by PYY on osteoblast activity. Therefore, the overexpression of PYY exhibited a suppressive effect on osteoblasts while stimulating osteoclasts, leading to a decline in bone mass. Conversely, the pharmacological inhibition of Y1R signaling elicited an enhancement in bone formation and a reduction in bone resorption, subsequently enhancing the overall bone microarchitecture in rats subjected to ovariectomy (Wong et al., 2012; Xie et al., 2020). Interestingly, PYY might additionally exert a modulatory influence on bone metabolism via its interaction with hypothalamic Y2Rs. Selectively conditioned deficient adult mice, germline Y2R-deficient hypothalamic mice, and hypothalamic-specific Y2RKO mouse models all showed increased bone formation. Although the number of osteoblasts remained unchanged, the rate of mineralization was higher, consequently culminating in an augmentation of the magnitude of bone trabeculae in Y2R KO mice. In addition to promoting bone formation, diminishing Y1R signaling in osteoblasts hampers glucose tolerance and curtails insulin secretion, elucidated by the diminished exudation of osteoglycin, a regulator of insulin action (Lee et al., 2018). Because modified levels of PYY are correlated with various metabolic disorders that also impact bone mass, PYY may potentially exert an influence on bone homeostasis.

### 7.2 PYY’s effect on bone metabolism in humans

The detrimental impacts of PYY on osteogenesis, as observed in the majority of preclinical investigations, receive further validation from observational inquiries revealing a negative correlation between heightened fasting PYY levels and a BMD in physically active premenopausal women and those afflicted by anorexia nervosa (Scheid et al., 2011; Utz et al., 2008). Low bone mass is prevalent in individuals with anorexia nervosa, as evidenced by a study investigating BMD in adult female patients diagnosed with this condition. The findings of this study unveiled a robust inverse relationship between the nocturnal mean levels of the PYY and BMD measurements taken at various skeletal sites, including the spine, femoral neck, distal radius, total hip joint, and radius (Utz et al., 2008). This is thought to be associated with lower mechanical load, growth hormone resistance, and hypogonadal function (Fazeli and Klibanski, 2018). Likewise, in young female athletes with amenorrhea, the increase of PYY was negatively correlated with P1NP, which further supported the effect of PYY on bone homeostasis (Russell et al., 2009). Bariatric surgery, such as the Roux-en-Y gastric bypass, is correlated with heightened bone turnover (CTX-Ⅰ) and sustained bone diminishment that endures until post-weight stabilization (Lindeman et al., 2018). The factors contributing to bone loss after bariatric surgery are believed to encompass unloading, alterations in calcitonin, and possibly expressed in, modifications in the secretion of gut hormones involving higher levels of PYY (Misra and Bredella, 2021). Although the administration of DPP-4 inhibitors, such as selegiline, presents potential for elevating PYY(1-36) levels (Aaboe et al., 2010), which may increase Y1R signaling resulting in decreased osteoblast activity, registry-based studies have failed to demonstrate any adverse effects of DPP-4 inhibitors on bone (Starup-Linde et al., 2017). PYY(3-36) analogs are currently undergoing clinical development as drugs for weight management. Importantly, considering the preclinical evidence indicating that these compounds might hinder bone formation by engaging with YR2 within the hypothalamus, it becomes imperative to investigate the impact of PYY analogs on human osteoblasts both *in vitro* and *in vivo*.

### 7.3 Relationship between PYY, GM, and bone metabolism

According to the previous section, PYY is considered to be a negative regulator of bone metabolism. The production of PYY is likewise regulated by the GM. It has been shown that ceftazidime (an anti-Gram-negative bacterial antibiotic) treatment significantly promotes PYY secretion in mice fed a high-fat diet (Rajpal et al., 2015). Butyrate produced by the GM metabolism strongly promotes PYY expression through the activation of Toll-like receptors (TLR) in L-cells (Larraufie et al., 2017). In addition, bile acids activate the GPCR of PP cells, and Takeda G-protein receptor 5 (TGR5) stimulates PYY release, whereas H_2_S produced by sulfate-reducing commensal bacteria in the colon inhibits TGR5-dependent PYY release involving the PLC-ε/calcium ion pathway (Bala et al., 2014).

Therefore, while it appears that PYY possesses formidable weight-reducing properties, exogenous PYY negatively regulates bone mass and strength in adults and has long-term deleterious side effects on bone, including increasing the risk of fracture, so care should be taken in treating obesity with PYY. At the same time, the promotion of PYY secretion by the GM should not be ignored, as it leads to further bone loss.

## 8 Conclusion and expectations

Osteoporosis is defined as a systemic bone disease characterized by decreased bone mass and deterioration of bone tissue microstructure, resulting in increased bone brittleness and fracture susceptibility (Rachner et al., 2011). Osteoporosis increases the risk of fracture, which is harmful and is one of the leading causes of disability and death in elderly patients. Osteoporosis-related fractures are becoming more common among older people, leading to a large number of bone-related diseases and increasing mortality and medical costs. With the aging of the global population, the incidence of osteoporotic fracture is still in a period of rapid growth. Given that the number of people with osteoporotic fractures is predicted to increase from 60 million to more than 120 million by 2050 (García-Gómez and Vilahur, 2020), and the cost of treating osteoporotic fractures is projected to reach at least $25 billion by 2025 (Camacho et al., 2021), the issue presents an escalating quandary for healthcare systems.

Fracture is one of the common complications in patients with osteoporosis, usually requires hospitalization and fixation, and may lead to further complications, such as infection and ischemic osteonecrosis. The recovery process is often slow and incomplete (Kanis, 2002; Kanis et al., 2018). Pathological fractures are caused by several endogenous factors (decreased estrogen levels) or exogenous factors (malnutrition) and are characterized by decreased bone mass and microstructural alterations. Due to the long-term nature of fracture recovery and the necessity of treatment, several drugs have been developed for osteoporosis. Based on the underlying mechanism of action, anti-osteoporosis drugs are categorized into bone resorption inhibitors, bone formation enhancers, double-acting drugs, and other mechanism drugs (Table 2).

**TABLE 2:** Current drugs for osteoporosis treatment and their mechanisms.

Type	Name	Mechanism
**ANTIRESORPTIVE**		
Bisphosphonates	Alendronate	Tightly binding to hydroxyapatite and directly altering osteoclast morphology, thereby inhibiting osteoclast activity and suppressing bone resorption
	Risedronate	
	Ibandronate	
	Zoledronic acid	
Estrogens/SERMs	Raloxifene	Combining with estrogen receptor α in bone tissue to inhibit the activity of osteoclasts, balance bone remodeling, and maintain the balance of bone metabolism
	Bardoxifene	
RANKL inhibitors	Denosumab	Blocking the binding of RANKL and RANK to inhibit the maturation of osteoclast precursors
Calcitonin	Salmon calcitonin	Inhibiting osteoclast activity and osteolysis
**ANABOLIC**		
PTH-related peptide (PTHrP)	Teriparatide	Promoting bone formation through intermittent activation of the PTH1 receptor
	Abaloparatide	
**OTHERS**		
Active vitamin D	Eldecalcitol	Acting on human osteoclast progenitor cells and inhibiting the formation of osteoclasts
Monoclonal antibody	Romosozumab	Upregulating the Wnt pathway to increase bone formation and blocking the RANKL pathway to decrease bone resorption
Strontium salt	Strontium ranelate	Acting on both osteoblasts and osteoclasts to promote bone formation and inhibit bone resorption
**UNDER RESEARCH**		
Monoclonal antibody	Blosozumab	Upregulating the Wnt pathway to increase bone formation and blocking the RANKL pathway to decrease bone resorption
	Setrusumab	
Cathepsin K inhibitor	Balicatib	Reducing the level of serum CTX-Ⅰ
	Odanacatib	

**Summary:** Current drugs for the treatment of osteoporosis are divided into promoting bone formation, inhibiting bone resorption, and other categories. Bisphosphonate is the most widely used drug in the treatment of osteoporosis. Teriparatide mimics endogenous PTH for bone formation and is the only marketed anabolic agent. Many studies on monoclonal antibodies, which have the dual functions of anti-absorption and promoting formation, have been published recently. Romosozumab is a monoclonal antibody approved by the FDA in 2019 that promotes bone formation by increasing the Wnt signaling pathway and inhibits bone resorption by antagonizing the RANKL signal pathway. Numerous novel drugs are currently being developed to target specific molecules involved in the bone remodeling process, such as cathepsin K inhibitors, an enzyme secreted from osteoclasts that is necessary during absorption. Balicatib was in Phase I clinical trials but was discontinued due to safety concerns as subjects developed scleroderma-like skin sclerosis. Odanacatib, which has entered Phase III clinical trials, has been shown to consistently increase lumbar and hip BMD and decrease serum CTX-Ⅰ levels. Another monoclonal antibody to sclerostin, Setrusumab, is currently in Phase II clinical trials. Single-dose and subsequent clinical trials have shown similar results to those of romosozumab, as well as a lack of parallelism between bone formation and resorption in the treatment of osteoporosis.

Romosozumab is a monoclonal antibody against osteostatin that not only inhibits the activity of osteostatin (sclerostin) and upregulates the Wnt signaling pathway but also blocks the RANKL pathway, antagonizes its negative regulation of bone metabolism, promotes bone formation, and inhibits bone resorption (Cosman et al., 2016). However, romosozumab carries the potential to increase the risk of heart attack, stroke, and cardiovascular death. Due to inadequate efficacy and insufferable adverse effects, the current drugs for the treatment of osteoporosis are limited, so there is an urgent need to develop specific molecules that can safely maintain bone homeostasis.

**FIGURE 4:**
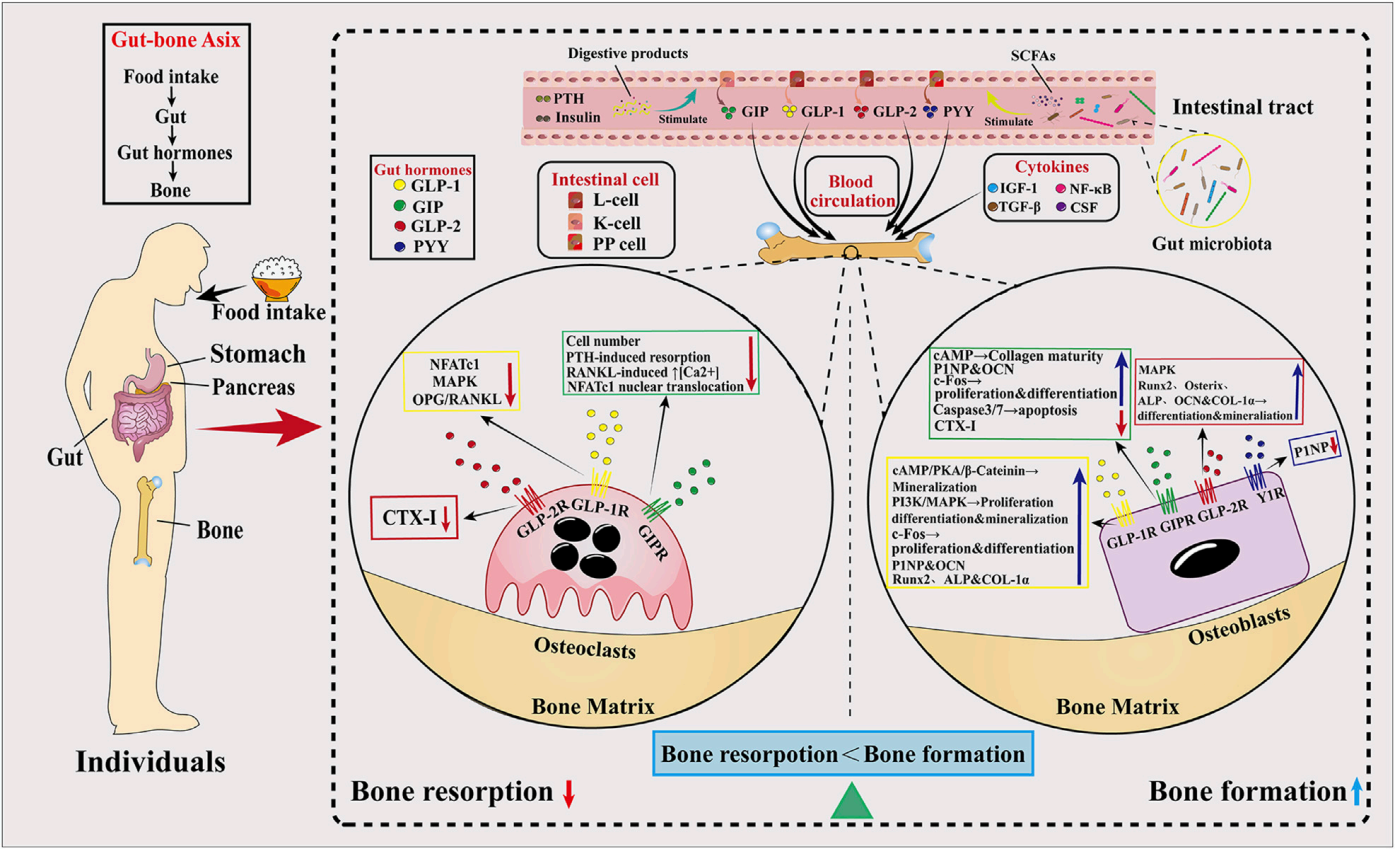
Mechanisms of incretin hormones’ effect on bone. The body’s response to food affects bone metabolism. First, the elevation of blood glucose levels subsequent to a meal stimulates the secretion of insulin, which in turn exerts its influence upon osteoclasts and triggers a reduction in bone resorption. More importantly, eating stimulates the secretion of incretin hormones from the intestine. Under the stimulation of nutrients, GLP-1, GLP-2, GIP, and PYY are secreted from the intestines and reach the bone through the blood circulation. Among them, GLP-1, GLP-2, and GIP bind to homologous receptors in osteoclasts, causing a decrease in the number of osteoclasts and a decrease in the osteoclast markers, CTX-Ⅰ, and the ratio of OPG/RANKL, and a decrease in PTH-induced bone resorption. On the other hand, GLP-1, GLP-2, GIP, and PYY act on homologous receptors in osteoblasts, activating intracellular MAPK, PKA, and PI3K pathways, promoting osteoblast proliferation and differentiation and mineralization, with elevated osteogenic markers P1NP and OCN (except for PYY) leading to osteogenic effects. Notably, the SCFAs produced by the GM also promote the secretion of GLP-1 and GLP-2, leading to increased bone formation. Overall, the osteogenic effect of gut hormones is greater than the osteoclastic effect, leading to increased bone formation.

Recent preclinical and clinical studies have shown that gut hormones may have a profound effect on bone remodeling and, ultimately, bone structure and strength (Figure 4). *In vivo* and *in vitro* studies in animals have shown that GIP mainly inhibits bone resorption, whereas GLP-1 promotes bone formation and inhibits bone resorption to enhance bone properties, but the relationship between GLP-1 and fractures remains controversial (Mabilleau et al., 2013; Aoyama et al., 2014; Pereira et al., 2015; Huang et al., 2023). In humans, GIP may inhibit bone resorption during intravenous infusion, and the antiresorptive effect appears to be more pronounced during hyperglycemia (Bergmann et al., 2019). Exendin-4 and liraglutide appear to be beneficial for bone reconstruction, and studies have mainly shown antiresorptive effects in animals. In contrast, the effects of GLP-1RAs on humans remain elusive. *In vitro* studies suggest that GLP-1 may promote bone formation in humans by affecting osteogenic progenitor cell differentiation, but there is a lack of similar studies on the effects of GLP-1 and GLP-1RAs on mature osteoblasts. GLP-1RA-stimulated bone blood flow seems to be very interesting and promising in osteoporosis and diabetic fractures. However, clinical data are still lacking, and so far, studies that have determined a relationship between GLP-1RA use and reduced fracture risk have been negative. Therefore, long-term clinical studies comparing the effects of different GLP-1RAs on bone are necessary. From the sparse data available, it appears that GIP, but not GLP-1, alters bone remodeling in humans. The physiological effects of GIP, GLP-1, and GLP-1RAs on bone metabolism have not yet been determined, and studies are needed to examine the effects of gut hormone on human osteoblast differentiation and osteoclast activity, including their potential interactions with Wnt and NF-kB signaling. Importantly, the potential effects of GLP-1RAs on osteoblasts *in vitro* and *in vivo* may differ significantly in preclinical and clinical studies due to differences in GLP-1R expression in animals and humans. Although there is evidence to support a direct effect of certain gut hormones on bone resorption and bone formation, a number of questions, including those previously raised in this review, remain unclear. Gut hormones are secreted at different time points after nutrient intake and at different levels, depending on the composition of the food, suggesting that the effects of these hormones on osteoblasts may depend on the sequence of exposure to the hormones and the source of stimulation available. Furthermore, the combinatorial effects of gut hormones are lacking. Thus, although several studies have been performed, additional multicenter randomized controlled trials are needed to analyze bone tissue at different sites in patients with different metabolic statuses and treated with different versions of GLP-1RAs. Elucidation of the specific processes and associated molecular pathways of GLP-1 will help elucidate the effects of GLP-1 on bone metabolism and its mechanisms.

In addition to gut hormones, there is a close and complex link between metabolites associated with the GM and bone metabolism. On the one hand, the GM can be directly or indirectly involved in the regulation of bone mass through the regulation of host metabolism, calcium absorption, hormone levels, and the immune system. On the other hand, the metabolites related to the GM can also stably and effectively reflect the influence of the GM on bone metabolism. They are expected to be a new potential target for the prevention and treatment of osteoporosis. Among them, SCFAs are considered promising specific targets for future bone metabolism interventions and deserve further in-depth studies. Interventions such as intake of probiotics and prebiotics, adjustment of dietary structure, and fecal microbiota transplantation (FMT) can improve the composition and abundance of the GM and its associated metabolites to varying degrees and further regulate the above links and pathways, providing new ideas for osteoporosis prevention and treatment. However, the translation of GM-related metabolites from animal studies to clinical application faces challenges, such as the selection of metabolites, the safety and efficacy of application, and the determination of the appropriate application time and dosage (Liao et al., 2021; Smirnov et al., 2016), which require more attention and effort from researchers in the future. With the rapid development of modern and emerging technologies, researchers are exploring more deeply the metabolites and pathways related to the promotion and regulation of bone metabolism and the maintenance of bone health (Chakrabarti et al., 2022; Li et al., 2022; Sato et al., 2021), thus providing more ideas and methods for the prevention and treatment of osteoporosis, the improvement of bone metabolism-related diseases, and the maintenance of bone health in the future.

In conclusion, the gut plays an important role in maintaining bone homeostasis, and a variety of gut-derived factors regulate bone metabolism. Despite significant progress in the development of drugs for the prevention and treatment of osteoporosis, there is limited research on the role of gut hormones in bone metabolism, the exact mechanism of their treatment of osteoporosis is not yet clear, and real and potential side effects pose challenges to the development of targeted drugs. In addition, due to the lack of sufficient clinical data, larger samples and longer-term clinical studies are needed to confirm the effects of enterokinetic hormones on bone metabolism and to explore the exact mechanisms involved. Because GPCR is often a good drug target, elucidating the exact mechanism of the effect of incretin hormones on bone metabolism is crucial for the development of new targeted drugs in the future.

## Abbreviations

ALP, alkaline phosphatase; BMC, bone mineral content; BMD, bone mineral density; BMP, bone morphogenetic protein; BMU, basic multicellular unit; COL-1, collagen type I; CTX-I, C-terminal telopeptide of type 1 collagen; DPP-4, dipeptidyl peptidase-4; GLP-1, glucagon-like peptide-1; GLP-2, glucagon-like peptide-2; GIP, glucose-dependent insulinotropic polypeptide; GM, gut microbiota; IGF-1, insulin-like growth factor-1; MMP, metalloproteinases; MCP-1, monocyte chemoattractant protein-1; M-CSF, macrophage colony-stimulating factor; NFATc1, nuclear factor of activated T cells 1; OCN, osteocalcin; OPG, osteoprotegerin; OPN, osteopontin; OSX, osterix; TGF-β, transforming growth factor-β; P1NP, type I procollagen N-terminal propeptide; PTH, parathyroid hormone; PYY, peptide YY (or peptide tyrosine tyrosine); RANK, receptor activator of NF-κB; RANKL, receptor activator of NF-κB ligand; RUNX2, runt-related transcription factor 2; SCFAs, short-chain fatty acids.
